# Compartmentalization of galactan biosynthesis in mycobacteria

**DOI:** 10.1016/j.jbc.2024.105768

**Published:** 2024-02-16

**Authors:** Karin Savková, Maksym Danchenko, Viktória Fabianová, Jana Bellová, Mária Bencúrová, Stanislav Huszár, Jana Korduláková, Barbara Siváková, Peter Baráth, Katarína Mikušová

**Affiliations:** 1 Department of Biochemistry, Faculty of Natural Sciences, Comenius University in Bratislava, Bratislava, Slovakia; 2 Institute of Chemistry, Slovak Academy of Sciences, Bratislava, Slovakia

**Keywords:** cell wall, galactosyltransferases, GlfT2, *Mycobacterium smegmatis*, proteomics, radiolabeling assays, subcellular fractionation

## Abstract

Galactan polymer is a prominent component of the mycobacterial cell wall core. Its biogenesis starts at the cytoplasmic side of the plasma membrane by a build-up of the linker disaccharide [rhamnosyl (Rha) – N-acetyl-glucosaminyl (GlcNAc) phosphate] on the decaprenyl-phosphate carrier. This decaprenyl-P-P-GlcNAc-Rha intermediate is extended by two bifunctional galactosyl transferases, GlfT1 and GlfT2, and then it is translocated to the periplasmic space by an ABC transporter Wzm-Wzt. The cell wall core synthesis is finalized by the action of an array of arabinosyl transferases, mycolyl transferases, and ligases that catalyze an attachment of the arabinogalactan polymer to peptidoglycan through the linker region. Based on visualization of the GlfT2 enzyme fused with fluorescent tags it was proposed that galactan polymerization takes place in a specific compartment of the mycobacterial cell envelope, the intracellular membrane domain, representing pure plasma membrane free of cell wall components (previously denoted as the "PMf" domain), which localizes to the polar region of mycobacteria. In this work, we examined the activity of the galactan-producing cellular machine in the cell-wall containing cell envelope fraction and in the cell wall-free plasma membrane fraction prepared from *Mycobacterium smegmatis* by the enzyme assays using radioactively labeled substrate UDP-[^14^C]-galactose as a tracer. We found that despite a high abundance of GlfT2 in both of these fractions as confirmed by their thorough proteomic analyses, galactan is produced only in the reaction mixtures containing the cell wall components. Our findings open the discussion about the distribution of GlfT2 and the regulation of its activity in mycobacteria.

Pathogens from *Mycobacterium* spp. cause devastating infectious human diseases, among which tuberculosis (*Mycobacterium tuberculosis*) and leprosy (*Mycobacterium leprae*) are the most well-known (1, 2). Recently, mycobacterioses caused by non-tuberculous mycobacteria (such as *Mycobacterium abscessus*) manifesting as severe lung, skin, and mucosal infections, were reported to be on the rise (3). The common feature of these diseases is their resistance to conventional antibiotics, their persistence and an extremely long time required for the treatment (1, 2, 3). Among the reasons why it is so difficult to cure mycobacterial infections is a unique cell envelope of these pathogens—an extraordinary sturdy, but dynamic structure providing an efficient protection against the hostile environment in the human host and facilitating communication with the immune system of the infected person (4).

From an ultrastructural point of view, the mycobacterial cell envelope is diderm, containing a conventional plasma membrane and a specific outer membrane, or mycomembrane, which are separated by a periplasm (5). The inner leaflet of the mycomembrane is formed by specific long-chain (up to C_100_) 2-alkyl 3-hydroxylated fatty acids, designated as mycolic acids. They are part of the so-called cell wall core–a covalently linked complex of peptidoglycan, heteropolysaccharide arabinogalactan, and mycolic acids that esterify last and penultimate arabinoses at the nonreducing ends of branched arabinan chains. The inner layer of the mycomembrane is complemented with a range of mycobacteria-specific extractable lipids, as well as conventional phospholipids, in the outer leaflet (5).

A linear galactan polymer, composed of about 20 to 30 galactoses in rather uncommon furanose forms (Gal*f*), makes a critical component of the mycobacterial cell wall core (6). It is attached to the peptidoglycan proper *via* the linker disaccharide [rhamnosyl (Rha) – N-acetyl-glucosaminyl (GlcNAc) phosphate], which also serves as a primer for initiation of galactan synthesis (7). This pathway uses a lipid carrier, decaprenyl-phosphate, as an acceptor substrate, on which the whole galactan is built at the cytoplasmic side of the plasma membrane. The first steps of the pathway are carried out by N-acetylglucosaminyl phosphate transferase WecA (8, 9) and rhamnosyl transferase WbbL (10, 11). They produce a lipid-linked intermediate, decaprenyl-P-P-GlcNAc-Rha (glycolipid 2, GL2), which is extended by two bifunctional galactosyl transferases, GlfT1 and GlfT2 (12). GlfT1 initiates the galactan synthesis by attaching the first and the second Gal*f* to GL2 by β(1–4) and β(1–5) glycosidic bonds, respectively. GlfT2 then takes over, producing decaprenyl-P-P-GlcNAc-Rha-Gal*f*_x_ - a heterogenous population of molecules termed lipid-linked galactan (LLG) polymer, presumably containing 20 to 30 alternating β(1–6) and β(1–5) linked Gal*f* (12). It should be noted that the actual size of galactan translocated across the plasma membrane is not currently known, as it is not possible to obtain this material from mycobacteria (*Msmeg*) under standard conditions (13). The Gal*f* building blocks are provided by UDP-galactopyranose mutase Glf, which converts UDP-galactopyranose to UDP-Gal*f* serving as a donor substrate for both GlfT1 and GlfT2 (14). The galactan polymer is translocated from the cytoplasmic face of the plasma membrane to the periplasm by a recently discovered ABC transporter Wzm-Wzt (13). The cell wall core synthesis is then finalized by the action of an array of arabinosyl transferases, mycolyl transferases, and ligases that catalyze an attachment of the arabinogalactan polymer to peptidoglycan through the linker region (15) (Fig. 1).

**Figure 1:**
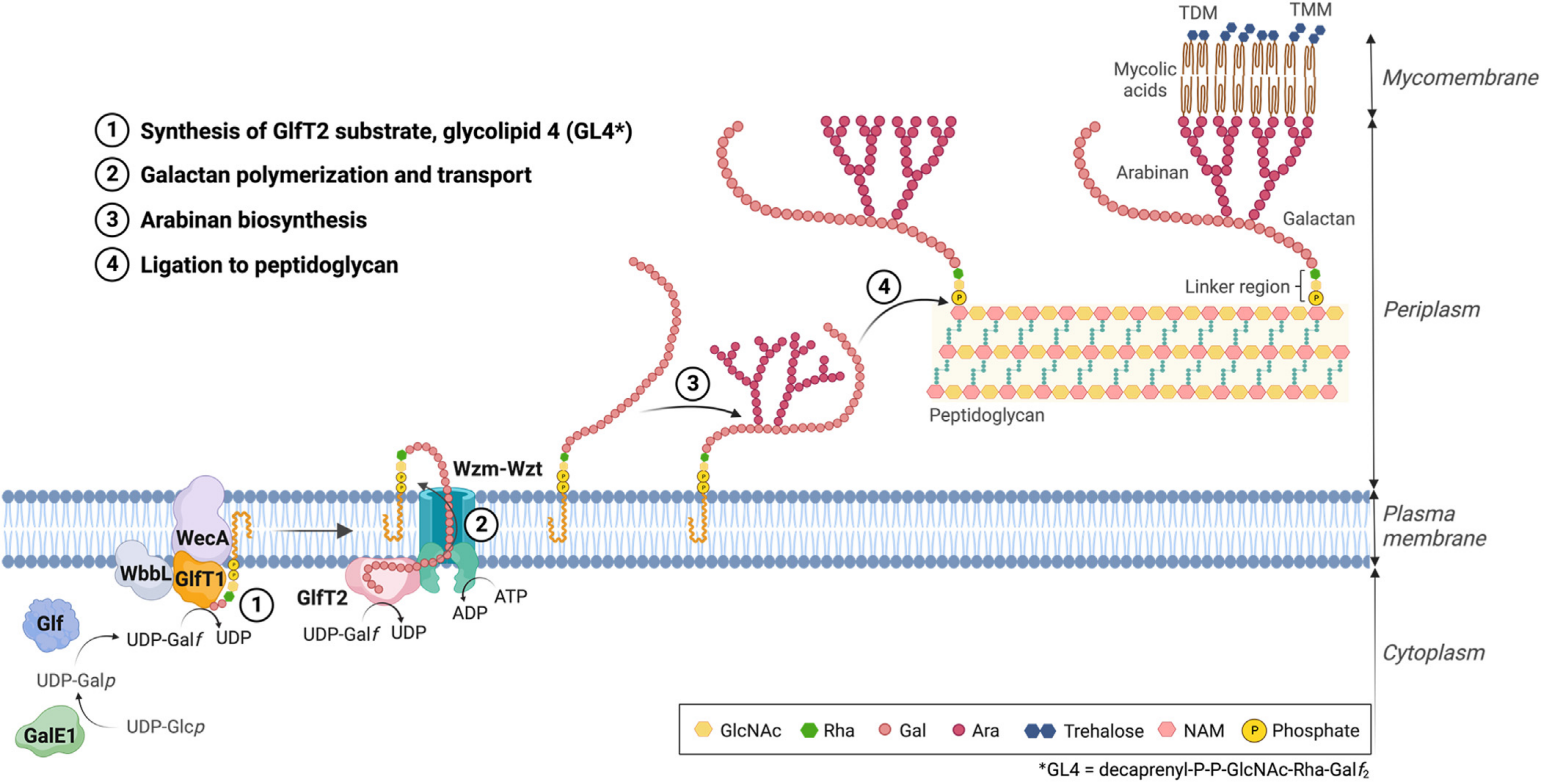
**Schematic representation of the mycobacterial cell wall core organization, biosynthesis of arabinogalactan and its attachment to peptidoglycan.** GalE1 – UDP-glucose 4-epimerase, Glf – UDP-galactopyranose mutase, WecA – N-acetylglucosaminyl phosphate transferase, WbbL – rhamnosyl transferase, GlfT1 – initiating galactosyl transferase, GlfT2 – a polymerizing galactosyl transferase, Wzm-Wzt – an ABC exporter for the galactan polymer. Ara, arabinose; Gal, galactose; GlcNAc, N-acetylglucosamine; NAM, N-acetylmuramic acid; Rha, rhamnose; TDM, trehalose dimycolates; TMM, trehalose monomycolates; UDP-Gal*f*, UDP-galactofuranose; UDP-Gal*p*, UDP-galactopyranose; UDP-Glc*p*, UDP-galactopyranose. The image was created with BioRender.com.

Fluorescently labeled versions of GlfT2 (MSMEG_6403) with N terminally fused mCherry are widely used as a specific marker for an intracellular membrane domain (IMD) in *Mycobacterium smegmatis* mc^2^155 (*Msmeg*) (16). The presence and physiological relevance of two distinct membrane domains in mycobacteria, was first proposed by Morita *et al.*, in 2005 (17). They observed that during the separation of the *Msmeg* cell lysate on sucrose gradient, two functionally different membrane fractions are formed. The lipidic material found in the less dense region of the sucrose gradient did not contain cell wall constituents, and it was originally denoted as the PMf domain (*i.e.*, plasma membrane free of cell wall). In the material collected from the denser region, the typical components of the cell wall core (*e.g.*, galactose), as well as a spectrum of conventional phospholipids, were present; so, this fraction was classified as plasma membrane tightly associated with the cell wall (PM-CW) (17). In a follow-up work by Hayashi *et al.* published in 2016 (18), a detailed proteomic characterization of PMf revealed that GlfT2 is a highly abundant enzyme in this domain, and it was chosen as a tool to refine its proteomic composition. Specifically, a transgenic strain of *Msmeg* was constructed, in which the endogenous *glfT2* was replaced with an mCherry (mC) fusion gene, hemagglutinin (*HA*)-*mC-glfT2*. The strain grew normally despite the exchange of the native and essential *glfT2* for the recombinant tagged version of GlfT2. The presence of HA-mC-GlfT2 was, indeed, confirmed in PMf, as expected. Next, the proteome of this fraction was examined in the sample obtained by antiHA immunoprecipitation and compared to identically treated protein preparation from the WT *Msmeg*. This experiment confirmed an enrichment with the selected proteins initially found in PMf proteome, such as mannosyl transferase PimB (MSMEG_4253), phosphatidylserine decarboxylase Psd (MSMEG_0861), polyprenol-monophosphomannose synthase Ppm1 (MSMEG_3859), glycosyltransferase Gtf1 (MSMEG_0389), geranylgeranyl reductase (MSMEG_2308), dihydroorotate dehydrogenase PyrD (MSMEG_4198), and a putative membrane protein (MSMEG_1944) (18). For five of these proteins (GlfT2, Gtf1, MSMEG_2308, PyrD, and MSMEG_1944), their localization to PMf was also confirmed by the construction of C-terminal HA fusions and monitoring HA-tags in the sucrose gradient fractions by Western blotting. Overall, based on comprehensive proteomic and lipidomic data, PMf was proposed to represent a distinct and metabolically active membrane domain serving as an organizational center for the biosynthesis of selected metabolites, especially those that are critical for the biosynthesis of the mycobacterial cell envelope, and it was renamed to IMD (19, 20).

Despite the reported high abundance of GlfT2 in the cell wall-free IMD, previous experiments monitoring the galactan biosynthesis in mycobacteria indicate that it requires the presence of cell wall components (21, 22). Driven by these initial observations, in this work, we examined the activity of the galactan-producing cellular machine in the thoroughly characterized cell wall-containing and cell wall-free plasma membrane fractions from *Msmeg* using the enzyme assays with radioactively labeled substrate, UDP-[^14^C]-galactose (UDP-[^14^C]-Gal), as a tracer. In addition, we assessed the distribution of polymerizing galactosyl transferase GlfT2 in these subcellular fractions and found that despite a high abundance of the GlfT2 protein in both of them, galactan is produced only in the reaction mixtures containing the cell wall components.

## Results

### GlfT2 is an abundant protein of the cell wall-containing and cell wall-free membrane fractions

To monitor the distribution of the galactan-synthesizing machinery in *Msmeg* we modified subcellular fractionation protocol, which we previously used for enzymology studies focused on the biosynthesis of the components of the mycobacterial cell wall core. Specifically, we simplified the preparation of the cell wall-containing fraction compared to our original procedure, which included separation of the 23,000*g* pellet obtained by centrifugation of the lysed bacteria in 60% Percoll and subsequent repeated washings of the material collected from the density gradient to remove Percoll. This fraction was referred to as “P60“or “cell envelope fraction” (CEF), and it comprised fragments of the cell wall and plasma membrane (21).

**Figure 2:**
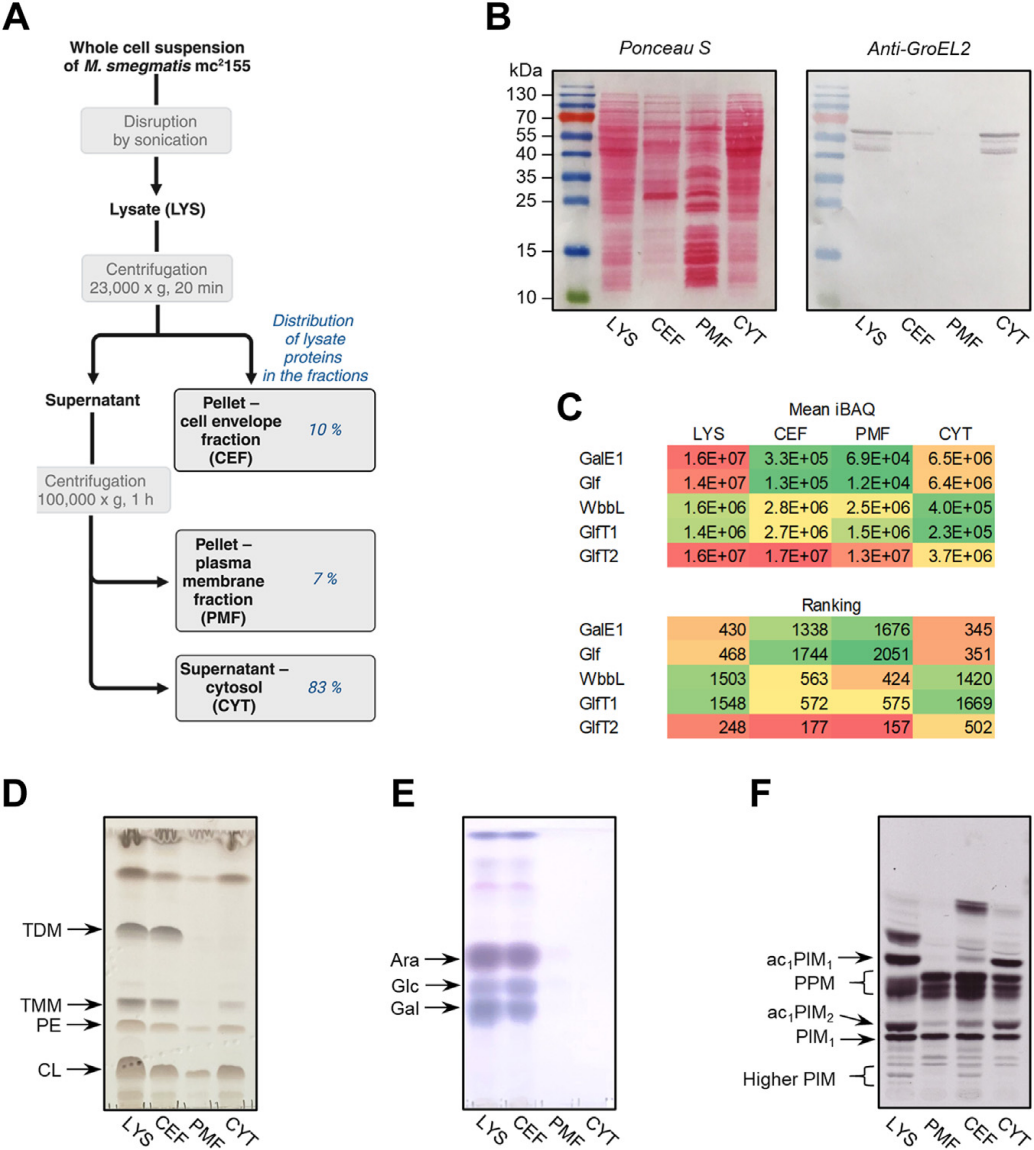
**Characterization of *Msmeg* subcellular fractions obtained by differential centrifugation.***A*, a schematic representation of the lysate fractionation by differential centrifugation. Cell envelope (CEF) and plasma membrane (PMF) fractions were resuspended in one quarter of the original volume of the centrifuged sample. The distribution of the lysate protein among the fractions represents an average from five independent experiments (Fig. S1*A*). *B*, protein analysis by SDS-PAGE and Western blotting. Aliquot volumes of the samples were analyzed, but the lysate and cytosol fractions were 10× diluted. *Left panel*: proteins were visualized with Ponceau S; *right panel:* immunodetection with anti-GroEL2 antibodies and alkaline phosphatase-conjugated secondary antibodies. *C*, mean iBAQ quantification values and rankings (protein abundance order within the fraction) globally color-coded in *red*-*yellow*-*green* (high-medium-low) scale for detected proteins involved in galactan biosynthesis (GalE1 – UDP-glucose 4-epimerase, Glf – UDP-galactopyranose mutase, WbbL – rhamnosyl transferase, GlfT1 – initiating galactosyl transferase, GlfT2 – polymerizing galactosyl transferase). Data from three independent biological replicates are shown in Dataset 1. *D*, TLC analysis of the lipid aliquots separated in CHCl_3_/CH_3_OH/H_2_O (20:4:0.5). The lipids were visualized with the cupric sulfate reagent. *E*, monosaccharide composition of insoluble pellets. The pellets were hydrolyzed with 2 M trifluoroacetic acid, and the released monosaccharides were separated by TLC in ethyl acetate/pyridine/glacial acetic acid/H_2_O (6:3:1:1). The monosaccharides were visualized with the *α*-naphthol stain. The bands were marked according to the positions of the monosaccharide standards comigrated with the samples. *F*, examination of mannolipid synthesis. TLC analysis of products from enzyme reactions performed with the fraction aliquots and GDP-[^14^C] Man. The extracted lipids were analyzed by TLC in CHCl_3_/CH_3_OH/NH_4_OH/H_2_O (65:25:0.5:4) and visualized by autoradiography. Panels *B*, *D*, *E*, and *F* are representative images from at least three independent biological replicates. Ara, arabinose; CEF, cell envelope fraction; CL, cardiolipin; CYT, cytosol; Gal, galactose; Glc, glucose; LYS, lysate; PE, phosphatidyl ethanolamine; PIM, phosphatidylinositol mannosides; PMF, plasma membrane fraction; PPM, polyprenylphosphomannoses; TDM, trehalose dimycolates, TMM, trehalose monomycolates.

In our current protocol, *Msmeg* were grown to the defined cell density (OD_600_ ∼ 1), disrupted by sonication and as the first step, the lysate was subjected to centrifugation at 23,000*g*, as before. However, the cell wall-containing CEF was obtained simply by resuspending 23,000*g* pellet in buffer A and centrifugation under the same conditions to remove the majority of the contaminating supernatant material. The pellet obtained by ultracentrifugation of 23,000*g* supernatant at 100,000*g* represents the plasma membrane fraction (PMF), while 100,000*g* supernatant corresponds to cytosol (Fig. 2*A*).

The purpose for this modification was to minimize the loss of material, so that we could quantify the distribution of the total proteins and lipids present in the lysate into these fractions, which were then used for the enzyme assays. We suspended the pellets of CEF and PMF fractions in volumes equal to one-quarter of the original volumes of the centrifuged samples (the lysate for CEF and 23,000*g* supernatant for PMF, respectively). This was taken into account in all subsequent analyses, in which we refer to “aliquots” of the samples. Thus, “aliquots” of CEF and PMF were the sample volumes corresponding to one-quarter of the volumes taken for lysate and cytosolic fractions.

Quantitative analysis of proteins in the obtained subcellular fractions revealed that the largest proportion of proteins is found in the cytosol (∼83%), followed by CEF (∼10%) and PMF (∼7%) (Figs. 2*A* and S1*A*). Qualitative differences between these fractions were apparent from SDS-PAGE profiles (Fig. 2*B*), and they were thoroughly inspected by proteomic analyses (Dataset 1). The quantitative distribution and ranking of the key proteins involved in the galactan polymer biosynthesis—UDP-glucose 4-epimerase GalE1 converting UDP-glucose to UDP-galactopyranose, UDP-galactopyranose mutase Glf, rhamnosyl transferase WbbL, an initiating galactosyl transferase GlfT1. and a polymerizing galactosyl transferase GlfT2 within the obtained fractions is shown in the Fig. 2*C*. The GalE1 and Glf enzymes acting on soluble sugar-nucleotide substrates were found primarily in the cytosol, as expected, and the three enzymes, WbbL, GlfT1, and GlfT2, employing the lipidic (decaprenyl-P-based) substrates were found enriched in both PMF and CEF (Fig. 2*C*). High ranking of GlfT2 in the lysate proves that the protein is abundant not only in PMFs as reported previously (18, 23), but also in the context of the total proteome of *Msmeg*. The locations of GalE1, WbbL, GlfT1, and GlfT2 were also examined by the analysis of the distribution of the N terminally His-tagged recombinant proteins overproduced in *Msmeg* (Fig. S2). Recombinant GalE1 was found primarily in the cytosol, while signals for His-tagged WbbL, GlfT1, and GlfT2 supported their association with PMF and CEF, although PMF was clearly the preferential location of recombinant GlfT2. Traces of the recombinant GalE1 were also found in CEF, which suggests its minor cross-contamination with cytosol (Fig. S2). This was also confirmed by observing a signal for a cytoplasmic marker, GroEL2 (24, 25), in this fraction (Fig. 2*B*). Examination of the distribution of the proteins characteristic for IMD and PM-CW between PMF and CEF by the mass spectrometry proved that PMF is enriched in several prominent proteins found in IMD, such as mannosyl transferase PimB (3.6×), phosphatidylserine decarboxylase Psd (1.5×), glycosyl transferase Gtf1 (1.8×), dihydroorotate dehydrogenase PyrD (1.9×), and a putative membrane protein MSMEG_1944 (5.3×), while in CEF highly abundant proteins of PM-CW, DivIVa family protein Wag31 and NADH dehydrogenase Ndh are over-represented (Fig. S1*B*). Among the characteristic IMD proteins, the quantities of Ppm1, GlfT2 and geranylgeranyl reductase MSMEG_2308 established by mass spectrometry were comparable or somewhat higher in a different domain, *i.e.*, cell wall-containing CEF. We also examined the distribution of the proteins from Mce (putative transporter), Msp (porin), and Ag85 (mycolyl transferase) families, which are characteristic for the mycomembrane (23) and they were found preferentially associated with CEF, as expected (Fig. S1*B*).

Next, we focused on the analysis of the lipid composition of the obtained fractions. Lipids were extracted from aliquots of lysate, PMF, CEF, and cytosol using mixtures of chloroform:methanol, and they were analyzed by TLC. As expected, we observed substantial amounts of trehalose monomycolates and trehalose dimycolates in CEF, but other standard membrane lipids such as cardiolipin, phosphatidylethanolamine, phosphatidylinositol, or phosphatidylinositol mannosides were present in both PMF and CEF (Figs. 2*D* and S1*C*). The relatively high content of lipids that was observed in the cytosolic fraction can be perhaps attributed to triacylglycerol-rich intracellular lipid inclusions that were described for different kinds of mycobacteria, including *Msmeg* (Fig. S1*C*) (26, 27).

To assess the presence of the monosaccharide cell wall core components in the prepared subcellular fractions, we subjected the delipidated pellets to a series of extractions, including incubation with hot 2% sodium dodecyl sulfate in phosphate-buffered saline to remove all soluble biomolecules. The resulting insoluble pellets were hydrolyzed with 2 M TFA, and the released monosaccharides were separated by TLC. This analysis revealed the presence of the key cell wall core monosaccharides arabinose and galactose, specifically in CEF (Fig. 2*E*), which is consistent with a high abundance of the major cell wall lipids, trehalose monomycolates, and trehalose dimycolates, in this fraction (Fig. 2*D*).

For further characterization of PMF and CEF we performed enzyme reactions with GDP-[^14^C]-mannose, since differential production of mannolipids served as one of the specific and defining features of IMD and PM-CW (17). As shown in Fig. 2*F*, CEF produced higher phosphatidylinositol mannosides (PIMs), as previously described for PM-CW (17).

Taken together, these data suggest that the PMF and CEF obtained by our modified protocol share several specific features with IMD and PM-CW fractions identified by Morita group (17, 18). Despite the previous recognition of GlfT2 predominance in IMD (18), our thorough quantitative proteomic analysis revealed that GlfT2 is a prominent and highly abundant component of both the IMD-like cell wall-free PMF and PM-CW-like cell wall-containing membrane fraction CEF (Figs. 2*C* and S1*B*).

### Galactan polymerization, catalyzed by mycobacterial enzyme fractions, requires the cell wall components

**Figure 3:**
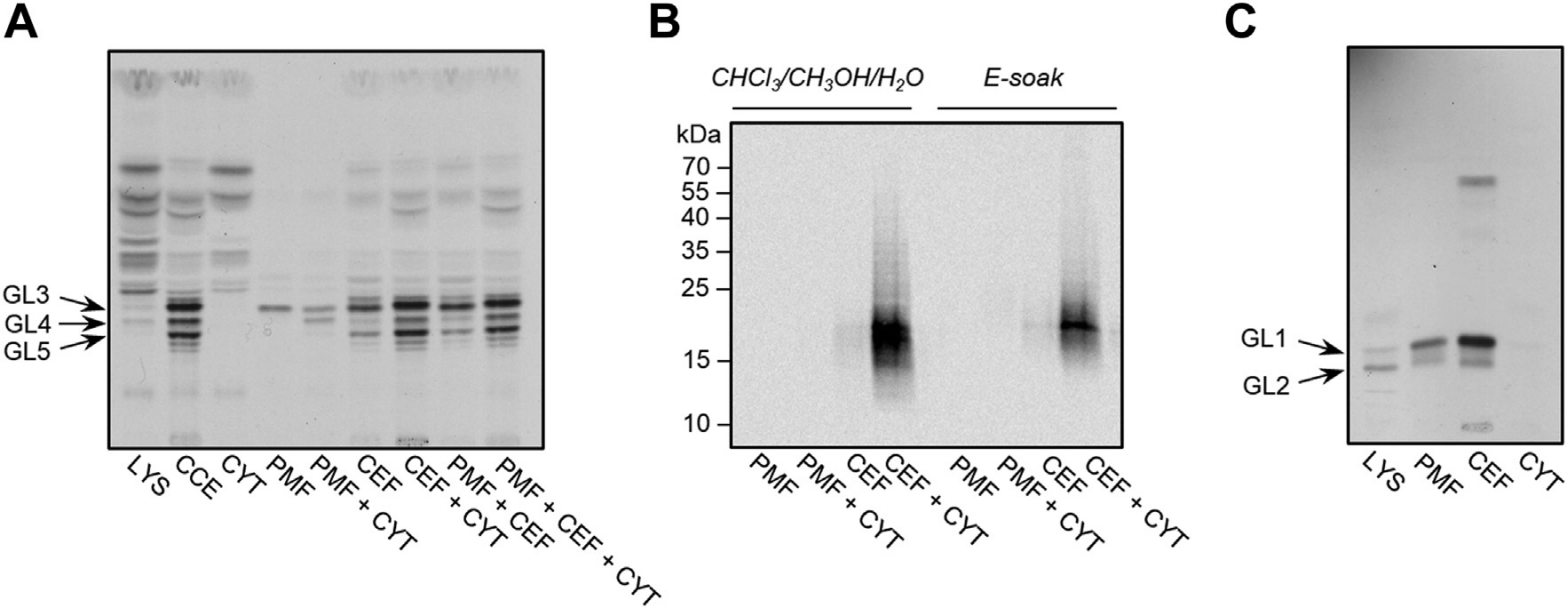
**Production of the lipid-linked [**^**14**^**C]-galactan and its precursors by subcellular fractions.** Fractions prepared from *Msmeg* by differential centrifugation served as the source of acceptor substrates and enzymes in the reaction mixtures, which were supplemented with UDP-GlcNAc, TDP-Rha and UDP-[^14^C]-Gal*p* (*A* and *B*), or [^14^C]-UDP-GlcNAc and TDP-Rha (*C*). *A*, TLC analysis of [^14^C]-labeled glycolipids separated in CHCl_3_/CH_3_OH/1 M ammonium acetate/NH_4_OH/H_2_O (180:140:9:9:23). *B*, analysis of the [^14^C]-LLG from CHCl_3_/CH_3_OH/H_2_O (10:10:3) and E-soak [H_2_O/C_2_H_5_OH/diethyl ether/pyridine/NH_4_OH (15:15:5:1:0.017)] extracts by SDS-PAGE followed by the transfer to a nitrocellulose membrane. *C*, TLC separation of [^14^C]-labeled GL1-2 in the solvent CHCl_3_/CH_3_OH/H_2_O/NH_4_OH (65:25:3.6:0.5). The radiolabelled reaction products were visualized by autoradiography (*A* and *C*) or phosphor imaging (*B*). Representative images from at least three independent biological replicates are shown. CCE, complete cell envelope (100,000*g* pellet of the lysate); CEF, cell envelope fraction; CYT, cytosol; GL, glycolipid; LLG, lipid-linked galactan; LYS, lysate; PMF, plasma membrane fraction.

The activity of the galactan-synthesizing machinery was examined by cell-free reactions, in which the prepared subcellular fractions served as the source of biosynthetic enzymes and their acceptor substrates. In addition to lysate, cytosol, PMF, and CEF, we also used the additional control, a complete cell envelope (CCE) fraction, which is a pellet from centrifugation of the cell lysate at 100,000*g* resuspended to the original volume of the centrifuged sample. We used aliquot volumes of the fractionated bacteria in all reactions. UDP-[^14^C]-Gal*p* served as a tracer and the reaction mixtures were supplemented with nonradioactive UDP-GlcNAc and TDP-Rha, which are necessary for the initiation of galactan biosynthesis by the N-acetylglucosaminyl phosphate transferase WecA and rhamnosyl transferase WbbL, respectively. In addition, PMF and CEF were spiked with cytosol to provide the source of Glf, which converts UDP-[^14^C]-Gal*p* to UDP-[^14^C]-Gal*f.* After the incubation, the reaction mixtures were extracted with chloroform/methanol to obtain glycolipids GL3-5 (decaprenyl-P-P-GlcNAc-Rha-Gal*f*_1-3_), followed by more polar solvents, CHCl_3_/CH_3_OH/H_2_O (10:10:3) and E-soak, releasing LLG polymer. As shown in the Fig. 3*A*, substantial amounts of glycolipids GL3-5 were produced in the reaction mixtures that contained cell walls, except for the lysate, due to an efficient randomization of UDP-[^14^C]-Gal*p* through its conversion to UDP-[^14^C]-Glc by the action of GalE1 enzyme. Evidently, GlfT2 activity was observed only in CCE and CEF supplemented with cytosol (Fig. 3, *A* and *B*).

While we cannot rule out the presence of some residual unlyzed cells in the CEF prepared by our simplified procedure (Fig. 1*A*), they are not expected to notably contribute to the observed galactan-producing activity of this fraction, primarily because the galactan producing machinery is intracellular and the charged UDP-[^14^C]Gal added to the reaction mixtures will not easily cross the cell envelope to reach the cytoplasm. However, we did examine the galactan polymer production in reactions mixtures, where the enzyme fractions were replaced by an aliquot of the whole cell suspension used for the lysate preparation. In this experiment, we used *Msmeg* transformed with pVV2, which is more efficient in galactan production compared to the WT *Msmeg*. Fig. S3 shows the profiles of the [^14^C]-glycolipids (Fig. S3*A*) and [^14^C]-LLG extracted with CHCl_3_/CH_3_OH/H_2_O (10:10:3) and E-soak solvents (Fig. S3*B*), which prove that production of [^14^C]-labeled products by the whole cells is, indeed, negligible compared to the fractionated cells.

To exclude the lack of GL2 serving as an initial acceptor substrate for galactan build-up in PMF, we used radioactive UDP-[^14^C]-GlcNAc as the tracer and supplemented the reaction mixtures with TDP-Rha to enable its production. TLC analysis of the radiolabeled products proved that GL2 was efficiently produced in both PMF and CEF (Fig. 3*C*).

These data showed that efficient galactan polymerization requires the presence of the cell wall components, which are provided by CEF obtained by the differential centrifugation procedure.

### Examination of the galactan polymerizing machinery in fractions obtained by sucrose density gradient confirms its requirement for the cell wall components

Since the standard means of preparation of IMD and PM-CW fractions is the breakage of mycobacteria by nitrogen cavitation, followed by low speed (2500–3220*g*) centrifugation and by separation of the supernatant on sucrose density gradient (17, 18), we decided to follow this procedure and examine galactan polymerizing machinery and distribution of the proteins in the obtained fractions. As nitrogen cavitation apparatus used for the standard IMD and PM-CW preparation (17, 18) is not available to us, we initially investigated, if two different breakage methods affect the distribution of the monitored enzyme activities. We disrupted bacteria by sonication and by French Press, respectively, and then subjected the lysates to differential centrifugation, as described above (Fig. 1*A*). The results of the enzyme assays monitoring mannosyl transferase, as well as galactofuranosyl transferase activities were comparable for both breakage methods (Fig. S4).

**Figure 4:**
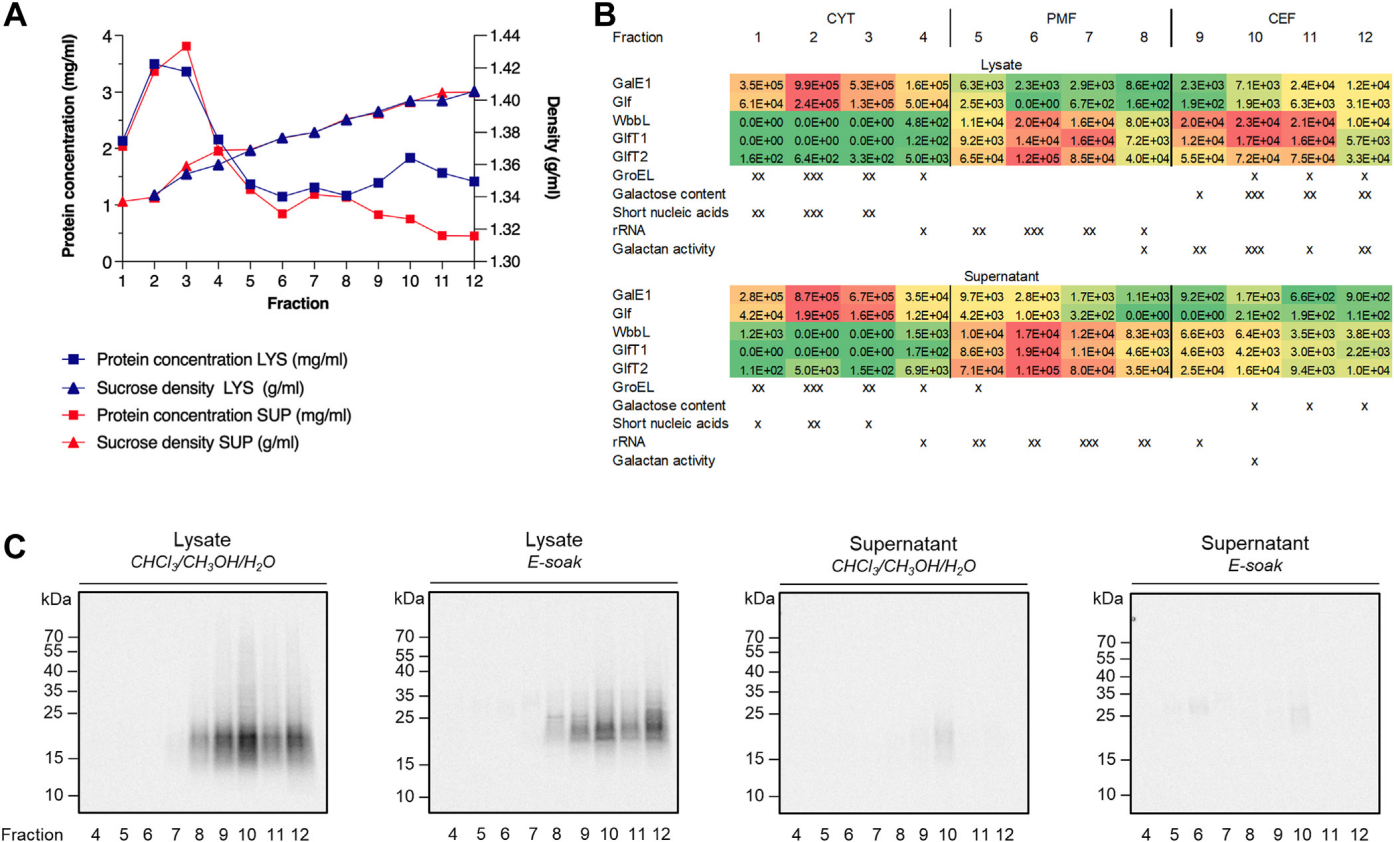
**Characterization of *Msmeg* fractions obtained by separation on the sucrose gradient.** The total cell lysate (LYS) and supernatant (SUP) from the low-speed centrifugation step were separated in 20 to 50% sucrose gradients. *A*, protein concentration and sucrose density across the fractions from LYS and SUP gradients. *B*, volume-normalized iBAQ quantification values distribution among fractions 1 to 12 color-coded in *red*-*yellow*-*green* (high-medium-low) scale for each detected protein involved in galactan biosynthesis (GalE1 – UDP-glucose 4-epimerase, Glf – UDP-galactopyranose mutase, WbbL – rhamnosyl transferase, GlfT1 – initiating galactosyl transferase, GlfT2 – polymerizing galactosyl transferase). Additional proteins are shown in Fig. S6. Manual estimation of selected parameters from Fig. S5 in arbitrary units 0 to 3 × (low-high). *C*, production of [^14^C]-LLG in enzyme reactions. Fractions 4 to 12 from sucrose gradients served as the source of acceptor substrates and enzymes in the reaction mixtures, which were supplemented with UDP-GlcNAc, TDP-Rha and UDP-[^14^C]-Gal*p*. [^14^C]-LLGs extracted by CHCl_3_/CH_3_OH/H_2_O (10:10:3) and E-soak [H_2_O/C_2_H_5_OH/diethyl ether/pyridine/NH_4_OH (15:15:5:1:0.017)] were analyzed by SDS-PAGE, followed by the transfer to a nitrocellulose membrane. Radioactive signals were detected by phosphor imaging. LLG, lipid-linked galactan.

For the next experiment, we chose French Press for the cell disruption. The first step, low-speed centrifugation of the lysate separated rather thick pellet (Fig. S5*A*), so we decided to perform the sucrose density gradient separation with the supernatant, as described, as well as directly with the bacterial lysate to avoid losing any material. All 12 fractions collected from the sucrose gradients were initially analyzed for the protein concentrations (Fig. 4*A*) and for the specific cytoplasmic and cell wall markers, respectively (Fig. S5). The strongest signal for the cytoplasmic marker GroEL was found in the first three fractions in both lysate and supernatant gradients (Fig. S5*B*); we therefore concluded that they correspond to cytoplasm. In the lysate gradient, a second, substantially smaller peak of GroEL signal was also found in the last three fractions. We observed minor carryover of the cytosol specifically to the CEF prepared by differential centrifugation described above (Fig. 1), which suggested that also the most dense fractions of the lysate gradient contain the cell wall material contaminated with cytosol. This was confirmed by the presence of the cell wall core marker, galactose, in these lysate fractions, although it was hardly detectable in the samples from the supernatant gradient (Fig. S5*C*). In the attempt to thoroughly characterize the fractions collected from the density gradients, we performed agarose electrophoresis to reveal the presence of nucleic acids. As shown in Fig. S5*D*, the pattern of separated nucleic acids was comparable for both lysate and supernatant fractions. They separated into three distinct regions—the first one corresponding to fractions 1 to 3, which contain small size nucleic acids, the second one corresponding to fractions 5 to 8 with the majority of rRNA, and the third one corresponding to fractions 9 to 12, which contain considerably smaller amounts of nucleic acids. At last, we performed proteomic analysis of the gradient samples (Fig. S6 and Dataset 1). The distribution of the proteins supported the division of the gradient fractions into three discrete regions. The cytosolic enzymes GalE1 and Glf were highly abundant in the least dense fractions 1 to 4. On the other hand, they contained minimal proportions of the monitored IMD-associated proteins discussed above (GlfT2, PimB, Ppm1, Gft1, MSMEG_2308, PyrD, Psd, and MSMEG_1944), and this was the case also for membrane-bound glycosyl transferases WbbL and GlfT1. The amounts of these proteins increased in the following region of middle sucrose density, comprised of fractions 5 to 8, which was especially prominent in the supernatant gradient fractions. Comparison of the overall quantities of IMD-associated proteins in the supernatant fractions 5 to 8 and in the most dense fractions 9 to 12 revealed their substantial enrichment in the former ones (GlfT2 – 5×, PimB – 11.9×, Gft1 – 2.6×, MSMEG_2308 – 3.5×, PyrD – 3.3×, Psd – 5.8×, MSMEG_1944 – 4.2×). However, this was not the case for the lysate gradient, where these proteins were found in substantially higher quantities in the fractions 9 to 12. Consequently, their enrichment in the combined fractions 5 to 8 considerably decreased (GlfT2 – 1.3×, PimB – 1.6×, Gft1 – 0.7×, MSMEG_2308 – 1.4×, PyrD – 1.2×, Psd – 1.5×, MSMEG_1944 – 1.8×) and the distribution of a selected set of these proteins (GlfT2, PyrD, Psd, MSMEG_2308) between the region 5 to 8 and 9 to 12 approached their allocation between PMF and CEF (Figs. S1*B* and S6). Finally, the lysate fractions 9 to 12 revealed high proportion of the mycomembrane associated proteins from Mce, Msp, and Ag85 families, as well as PM-CW associated proteins Ndh and Wag31, compared to fractions 5 to 8. Based on the above analyses summarized in Fig. 4*B*, we concluded that both lysate and supernatant gradient fractions separated into three comparable and distinct regions: cytoplasm (fractions 1–4), the cell wall-free PMF corresponding to IMD (fractions 5–8), and a cell wall-containing membrane fraction corresponding to PM-CW (fractions 9–12). However, in the supernatant gradient, the overall quantity of the monitored proteins and the cell wall marker galactose was lower in the most dense region, which we attribute to the loss of the material at the low-speed centrifugation steps (Figs. 4 and S5*A*).

Next, we examined the capacity of the individual fractions 4 to 12 to synthesize galactan. In this experiment, we omitted fractions 1 to 3, since galactan polymerization in these fractions is highly compromised by the abundance of GalE1 enzyme, which randomizes the UDP-[^14^C]-Gal*p* tracer. The peak of galactan production, *i.e.*, LLG precursors GL3-5 (Fig. S7*A*), but particularly LLG extracted with solvents CHCl_3_/CH_3_OH/H_2_O (10:10:3) and E-soak, for both supernatant and lysate gradients was found in the fraction 10 (Figs. 4*C* and S7*B*). However, for the supernatant gradient fractions this activity was much lower, compared to fractions 8 to 12 from the lysate density gradient, which were highly effective in synthesizing galactan precursors. This supports the conclusion that fractions 9 to 12 from the supernatant and lysate are qualitatively similar, and that the discrepancy in galactan-producing efficiency is caused by missing material, which was removed by low-speed centrifugation of the lysate. Nevertheless, as shown for the enzyme fractions obtained by differential centrifugation (Fig. 3), the galactan-producing machinery was found only in the cell wall containing fractions (Fig. 4*C*).

## Discussion

Since the discovery of the diderm structure of the mycobacterial cell envelope (28, 29), the question of the organization and coordination of the cell wall synthesizing machinery became pressing. Proteomic studies of different mycobacterial species revealed the presence of the enzymes involved in the synthesis of the components of the cell envelope in specific subcellular fractions, but this information was often contradictory. This can be exemplified by GlfT2, which was reported in the unique proteome of *M. leprae* PMF (100,000*g* pellet of the 27,000*g* supernatant of the lysed cells) (30), or in the cell wall fraction (22,000*g* pellet of the lysed cells) prepared from *Msmeg* (31). One of the reasons for these discrepancies could be that it proved to be rather challenging to obtain clean cytoplasmic, plasma membrane and cell wall fractions in mycobacteria (32). In the attempt to thoroughly characterize the composition of the native mycomembrane and the plasma membrane, Chiaradia *et al.* developed a procedure using the combination of differential and sucrose density centrifugation to obtain these two membrane fractions from *Msmeg* and *Mycobacterium aurum* in virtually pure forms (23). Among the most abundant differentially distributed proteins in these fractions were galactofuranosyl transferase GlfT2 and mannosyl transferase PimB found predominantly in the plasma membrane, and the proteins from Mce (putative transporter), Msp (porin) and Ag85 (mycolyl transferase) families were characteristic for the mycomembrane (23). However, while comprehensive proteomic and lipidomic analyses of these two fractions were instrumental for shedding light on the structure of the native mycomembrane, several studies showed that mycobacterial cell lysates tend to separate into two distinct plasma membrane-containing fractions by sucrose density gradient centrifugation—IMD, originally called PMf, and PM-CW (17, 18, 19, 20).

It attracted our attention that while GlfT2 has been widely used as an IMD marker, information about an association of galactan-producing metabolic pathway (or its portion) with this compartment is missing. As reported, it was impossible to find intermediates of galactan synthesis in the lipidome of this domain due to their transient character and expected very low abundance in the cells (18). In fact, LLG polymer was identified in mycobacteria only recently, in the *Msmeg* strain, with the silenced production of an ABC transporter Wzm-Wzt, which is responsible for its translocation to the periplasmic space (13).

Inspired by the earlier work focused on the evaluation of the distribution of PIMs and aminophospholipid synthesis in IMD and PM-CW fractions by cell-free assays (17), we decided to thoroughly characterize galactan-producing machinery in fractionated mycobacteria in a similar way.

Our initial presumption was that if the two membrane fractions (IMD and PM-CW) were distinct and physiologically relevant, it should be possible to obtain them by alternative means. We prepared the mycobacterial membrane fractions PMF and CEF by using different methods of cell breakage (French Press or sonication, *versus* originally used nitrogen cavitation) and differential, rather than sucrose density centrifugation. We showed that PMF and CEF, have several common features with IMD and PM-CW, respectively, the membrane domains originally described by Morita *et al.* (17, 18). Among them is the distribution of PIM synthesis. The early steps, the production of PIM_1_ and PIM_2_ took place both in PMF and CEF, while more glycosylated forms, PIM_4_-PIM_6_ were produced in CEF. Since the compartmentalization of the specific steps in PIM synthesis corresponds to the partitioning of the selected key proteins involved in this pathway between IMD and PM-CW (*i.e.*, PimB for lower PIMs in IMD and recombinant tagged PimE for higher PIMs in PM-CW (18)), it was proposed that galactan biosynthesis machinery would follow a similar scenario, *i.e.*, prevalent occurrence of the biosynthetic enzyme in the specific membrane domain would predict the site of the synthesis of the given metabolic intermediate (16). However, this does not appear to be the case for galactan synthesis, since despite high abundance of GlfT2 in PMF, its cell-free synthesis was found only in the enzyme fractions containing the cell wall components. Our current data are in accordance with earlier observations by us and the others, which showed that efficient cell-free galactan polymerization by fractionated mycobacteria required supplementation of the reaction mixtures with the cell wall containing enzyme preparations (21, 22). Analogous conclusion, *i.e.*, the requirement of the cell wall components for the efficient galactan synthesis, was also reinforced for the enzyme fractions obtained by sucrose density centrifugation.

This observation poses several important questions. The first one concerns the predictive value of the mere high abundance of GlfT2 in IMD for proposing this site as a compartment for galactan synthesis (16). Meniche *et al.* (33) studied the localization of the new cell wall synthesis in mycobacteria by investigation of the distribution of the fluorescently labeled enzymes MurG, GlfT2, and Pks13 involved in terminal cytosolic steps of peptidoglycan, arabinogalactan and mycolic acids and found that they colocalize and that they are enriched in subpolar region of the old pole (33), which is the preferential site of growth immediately after division (34, 35, 36). Time-lapse microscopy revealed that in addition to relatively stable localization at the pole, highly mobile forms of these enzymes are found along the lateral cell body and proposed that they are unlikely to be active (33). Consequently, pools of inactive and active forms of biosynthetic enzymes are expected to be present in bacteria and these could be distributed differentially between IMD and PM-CW domains. Moreover, we confirmed the enrichment with GlfT2 only in the cell wall-free PMFs prepared from the supernatant gradient, in which a substantial portion of the lysed material was missing due to low speed centrifugation step, challenging the value of GlfT2 as an IMD signature protein. The second question is related to mechanisms of regulation of the GlfT2 activity in mycobacteria, and specifically, why only GlfT2 present in the cell wall-containing fractions is active in the cell-free assays. The two obvious mechanisms that could be involved are the protein-protein interactions, or regulation by covalent modification, such as phosphorylation. Recently, we proposed that protein-protein interactions of GlfT2 with the galactan exporter Wzm-Wzt could be responsible for the galactan size regulation (13) and we are currently exploring this hypothesis. Although during the sucrose density centrifugation the transmembrane component of the transporter Wzm tends to be shifted toward CEF fractions (Fig. S6), in which we observed efficient galactan production, colocalization of the transporter with galactan synthesizing enzymes does not explain galactan producing activity in CEF, or the lack of it in PMF. As shown in Fig. S1*B*, in the samples obtained by differential centrifugation the presence of Wzm was evident not only in CEF, but also in PMF, which did not support galactan polymerization. Another attractive hypothesis regarding regulation of galactan producing machinery would be that GlfT2 phosphorylation state affects its activity. The recent analysis revealed that more than 80% of the *Mtb* proteome is O-phosphorylated (37). GlfT2 was shown to be the substrate for PknF, PknL, and PknK (37). Several reports on investigation of the roles of these protein kinases in mycobacterial physiology proposed that they affect the metabolism of the cell wall (38, 39, 40, 41). Verification of this hypothesis was beyond the scope of this study, but it should be noted that GlfT2 heterogeneously produced in *Escherichia coli* retains its enzyme activity (42, 43), so perhaps unphosphorylated GlfT2 is the enzymatically active form.

Compartmentalization of mycobacterial membranes between IMD and PM-CW and its dynamics were thoroughly studied (16, 44). They were found to be influenced by different physiological conditions, including the transition of the cells to the stationary phase or at starvation (20). Recently, it was proposed that membrane partitioning is driven by the cell wall (or rather peptidoglycan) polymerization catalyzed by transglycosylase domain of PonA2 (45), as well as by the production of tuberculostearic acid, or more specifically, by the action of S-adenosyl-L-methionine-dependent methyltransferase Cfa catalyzing the methylation of oleic acid (46). IMD was proposed as the growth pole-associated organizing center for synthesis of the cell envelope precursors (18), which “acts as a localized supply generator for cell envelope elongation” (16). Since this process occurs particularly at the poles (34), in rather long rod-shaped mycobacteria, much less plasma membrane would be present in the cell wall-free IMD than in the PM-CW domain. However, we noticed that in our experiments, in which we attempted to avoid loss of the material, PMF and CEF contain about 7% and 10% of the total protein lysate, respectively, so very likely fractions of the free plasma membrane and plasma membrane physically associated with the cell wall are quantitatively comparable. Or, in other words, IMD (corresponding to PMF) must be interspersed also along the long axis of the cells, questioning its physiological role as an organizing center for the cell wall synthesis, which takes place at/or close to the poles. Polar addition of the new cell wall material in mycobacteria was unequivocally proved by super-resolution microscopy, which visualized specific chemically modified precursors metabolically incorporated into the cell wall core. This can be exemplified by fluorescent D-alanine analogs applied for monitoring peptidoglycan synthesis (47), fluorescent trehalose-based probes used for imaging the mycomembrane (48, 49), or just recently described azide-containing analogs of farnesyl phosphoryl arabinose for labeling of mycobacterial arabinans by click chemistry (50). However, to our best knowledge, specific probes for monitoring galactofuranose incorporation into the cell wall core have not been described, which limits similar examination of galactan synthesis in mycobacteria.

An evidence for a physical connection of the portion of the plasma membrane with mycomembrane-containing cell wall core, which forms a discrete domain carrying out specific enzyme activities presented in this study, supports the original findings of Morita *et al.*, who, for the first time, described the mycobacterial PM-CW domain (17). Tight association of the plasma and outer membranes is well-documented in Gram-negative bacteria. It is ensured by elaborate protein complexes spanning the whole cell envelope (51, 52), but it is still enigmatic in mycobacteria. Only recently, a cryo-electron structure of the protein from Mce family provided the first example of the molecular scaffold connecting plasma membrane and mycomembrane (53). Another example might be the highly conserved mycobacterial protein Erp, a virulence factor playing role in maintaining the cell wall integrity (54).

To conclude—we developed a procedure for a lossless fractionation of mycobacterial lysates to the cytosolic, cell wall-free, and cell wall-containing PMFs and showed that cell-free galactan polymerization takes place in the last one of them. Recently, two genes encoding the enzymes from the galactan biosynthesis pathway, UDP-Gal mutase Glf and polymerizing galactosyltransferase GlfT2 were proposed to be highly vulnerable in *M. tuberculosis* (55). We believe that gaining insight into galactan polymerization in mycobacteria will help exploiting these enzymes as prospective targets for the tuberculosis drug development.

## Experimental procedures

### Cell material, fractionation, and analysis of fractions

#### Growth and breakage of mycobacteria

WT strain *M. smegmatis* mc^2^155 (*Msmeg*) or *M. smegmatis* mc^2^155 transformed with pVV2 (56) (*Msmeg*/pVV2) was inoculated from the glycerol stock into Sauton’s medium and cultivated at 37 °C with shaking (120 rpm) until the late-log phase. The starter culture was inoculated into LB medium supplemented with Tween 80 (0.05% v/v) and for *Msmeg*/pVV2 with kanamycin (20 μg/ml), and cultivated at 37 °C with shaking (120 rpm) until the OD_600_ ∼ 1. The cells were collected by centrifugation (5000*g*, 4 °C, 10 min) and washed once with buffer A consisting of 50 mM 3-(N-morpholino)propanesulfonic acid (MOPS) buffer (pH 7.9), 5 mM β-mercaptoethanol and 10 mM MgCl_2_ (3000*g*, 4 °C, 10 min). The cell pellet was suspended with buffer A as follows: 3 g of cells (wet weight) to which buffer A was added up to the final volume of 8 ml. The cells were routinely disrupted by sonication with Soniprep 150 (10 × with 60 s ON, 90 s OFF, amplitude 10) or by the French press (2 times, 0.8 kBar).

#### Fractionation by differential centrifugation

The lysate (10 ml) was centrifuged at 23,000*g* at 4 °C for 20 min. The pellet representing the CEF was washed once with buffer A and subsequently homogenized in the same buffer. The final volume of this CEF was adjusted with buffer A to 2.5 ml (4 × concentrated fraction compared to the original volume of lysate). Subsequently, 23,000*g* supernatant (7 ml) was centrifuged at 100,000*g* for 1 h at 4 °C. The pellet that represents the PMF was washed by centrifugation at 100,000*g*, 1 h at 4 °C and resuspended with buffer A to the final volume of 1.75 ml (4 × concentrated fraction compared to the original volume of 23,000*g* supernatant). The 100,000*g* supernatant represents the cytosol fraction. To prepare the CCE fraction, the bacterial lysate was centrifuged at 100,000*g*, 4 °C for 1 h, and the pellet was resuspended into the original volume of the centrifuged lysate with buffer A.

#### Fractionation by sucrose gradient

The lysate aliquot from *Msmeg* (3 ml) was centrifuged at 3200*g*, 10 min, 4 °C twice. To achieve the lysate densities and the sucrose gradient conditions approaching the protocols of Morita’s group (18), 1.24 ml of the supernatant or the lysate were mixed with 2.06 ml of buffer A (3.3 ml of the sample in total) and loaded onto 20 to 50% sucrose gradient (3.15 ml of 20%, 30%, 40%, and 50% sucrose solutions in buffer A; 12.6 ml in total). Ultracentrifugation was performed in Beckman ultracentrifuge tubes (#344061) at 170,000*g*, 4 °C for 6 h in SW32.1 Ti rotor on Optima L-100 XP ultracentrifuge. The sucrose density fractions (1.33 ml each) were collected from the top, resulting in 12 fractions in total. Protein concentration in individual fractions was measured by Pierce bicinchoninic acid protein assay kit, and sucrose density was measured by refractometry.

#### Lipid extraction and analysis

Aliquot amounts from the fractions obtained by differential centrifugation (1 ml of lysate and cytosol, and 250 μl of PMF and CEF) were subjected to extractions with 6 ml of CHCl_3_/CH_3_OH (1:2) and then with 3 ml of CHCl_3_/CH_3_OH (2:1) for 2 h and 1 h each at 56 °C. The extracts were combined, dried under a stream of nitrogen, and subjected to biphasic separation (2×) in CHCl_3_/CH_3_OH/H_2_O (4:2:1). The organic phases were dried under the nitrogen and dissolved in 100 μl of CHCl_3_/CH_3_OH/NH_4_OH/H_2_O (65:25:0.5:3.6). Equal amounts (4 μl) were used for thin layer chromatography on TLC silica gel 60 F_254_ in a mixture of CHCl_3_/CH_3_OH/H_2_O (20:4:0.5). Lipids were visualized by the cupric sulfate reagent [10% (w/v) CuSO_4_ in 8% (v/v) phosphoric acid].

#### Analysis of monosaccharide composition of the insoluble material

The pellets from lipid extraction steps were subjected to extractions with 1 ml 50% ethanol for 1 h at 100 °C (3×). Pellets were then extracted with 1 ml 2% SDS in PBS at 56 °C for 1 h (3×) and at 100 °C for 20 min (2×) to remove noncovalently linked material. SDS was washed away with 1 ml PBS (3×) and 1 ml 80% acetone (2×). The final pellets were left to dry under the nitrogen after second acetone wash to remove residual acetone, and subsequently hydrolyzed in 400 μl 2 M TFA at 120 °C for 2 h. After hydrolysis, the samples were dried and washed with several drops of methanol (2×) and H_2_O (2×). The dried hydrolysates were subjected to separation with 2 ml CHCl_3_/H_2_O (1:1). Equal volumes of water phase (24 μl) were analyzed on high-performance thin-layer chromatography silica gel plates developed twice in ethyl acetate/pyridine/glacial acetic acid/H_2_O (6:3:1:1). The monosaccharides were detected with an α-naphthol detection reagent [1% (w/v) α-naphthol and 5% (v/v) H_2_SO_4_ in ethanol].

For monosaccharide composition of insoluble material from sucrose density fractions, 500 μl of each fraction were extracted with 3 ml of CHCl_3_/CH_3_OH (1:2) and then twice with 2 ml of CHCl_3_/CH_3_OH (2:1) for 2 h each at 56 °C. The final delipidation step was carried out with 3 ml of CHCl_3_/CH_3_OH (2:1) for 5 h at 65 °C. After cooling, water (0.5 ml) was added to the sample to achieve the two-phase mixture of CHCl_3_/CH_3_OH/H_2_O (4:2:1). Upper and bottom phases formed after centrifugation (2500*g*, 10 min, 22 ˚C) were discarded. The resulting interphase was dried and hydrolyzed with 200 μl 2 M TFA at 120 °C for 2 h. Hydrolyzed samples were washed with methanol (3×) and water (3×) and subjected to extraction with 2 ml CHCl_3_/H_2_O (1:1). The water phase (50 μl) was analyzed by TLC as described above.

### Cell-free assays, extraction, and analysis of the reaction products

Enzyme activities were monitored in the reaction mixtures containing the following volumes of the obtained fractions: 80 μl of the whole cell suspension, 80 μl lysate, 20 μl PMF, 20 μl CEF, and 80 μl cytosol. Cytosolic fraction (5 μl) was added to selected reaction mixtures, when required. Reaction mixtures for monitoring galactan polymerization were supplemented with 2.35 mM NADH, 176.5 μM UDP-GlcNAc, 176.5 μM dTDP-Rha (Carbosynth), and 0.25 μCi UDP-[^14^C] Gal*p* (ARC, specific activity 55 mCi/mmol). Cell-free assays for monitoring the initial steps of galactan biosynthesis contained 176.5 μM dTDP-Rha and 0.125 μCi UDP-[^14^C] GlcNAc (ARC, specific activity 55 mCi/mmol). The reaction mixtures for monitoring mannolipid synthesis contained 0.01 μCi GDP-[^14^C] Man (ARC, specific activity 55 mCi/mmol). The volumes of all reaction mixtures were adjusted to 85 μl with buffer A. The reaction mixtures (40 μl) for examination of galactan biosynthesis in the sucrose gradient fractions contained 35 μl of the given fraction, 2.35 mM NADH, 176.5 μM UDP-GlcNAc, 176.5 μM dTDP-Rha (Carbosynth), 0.125 μCi UDP-[^14^C] Gal*p* (ARC, specific activity 55 mCi/mmol), and 2.5 μl of cytosol.

All reactions were incubated at 37 °C for 2 h and stopped by the addition of 1.7 ml of CHCl_3_/CH_3_OH (1:2). The mixture was centrifuged (10 min, 14,000*g*), the supernatant was collected and the pellet reextracted with 1.5 ml of CHCl_3_/CH_3_OH (2:1) by 20 min incubation on rotator at room temperature, followed by centrifugation (10 min, 14,000*g*). CHCl_3_/CH_3_OH extracts were combined, dried, and subjected to biphasic Folch wash (2×) with CHCl_3_/CH_3_OH/H_2_O (4:2:1). TLC analyses of the organic phases were performed on Silica gel F_254_ plates in appropriate solvent mixtures specified at the figure legends. The pellets were extracted with 1 ml of 0.9% NaCl in 50% CH_3_OH, 50% CH_3_OH, and 100% CH_3_OH, and 0.5 ml of CHCl_3_/CH_3_OH/H_2_O (10:10:3) and E-soak [H_2_O/C_2_H_5_OH/diethyl ether/pyridine/NH_4_OH (15:15:5:1:0.017)] as described before (21). LLG in the latter two fractions was analyzed by SDS-PAGE followed by blotting to nitrocellulose membrane. The radiolabeled reaction products were visualized by autoradiography (Biomax MR-1 film, Kodak at −80 °C), or phosphor imaging (Amersham Typhoon 5).

### Construction of *Msmeg* strains overexpressing *galE1*, *wbbL1*, *glfT1*, *glfT2*, and their analysis

#### Cloning of galE1, wbbL1, glfT1, and glfT2

The *MSMEG_6142* (*galE1*), *MSMEG_1826* (*wbbL1*), *MSMEG_6367* (*glfT1*), and *MSMEG_6403* (*glfT2*) genes were amplified from the genomic DNA of *M. smegmatis* mc^2^155 based on the oligonucleotide primers containing *Nde*I and *Hind*III restriction sites (Fig. S8). The amplified fragments were digested and ligated into the digested pVV2 vector (56) for constitutive expression of recombinant proteins with N-terminal hexahistidine motif in *M. smegmatis*.

#### Distribution of recombinant proteins in subcellular fractions

*Msmeg* strains transformed with plasmids pVV2-*galE1*, pVV2-*wbbL1*, pVV2-*glfT1*, and pVV2-*glfT2* were inoculated from the glycerol stocks into 5 ml LB medium with Tween 80 (0.05% v/v), kanamycin (20 μg/ml), and hygromycin (20 μg/ml) and cultivated at 30 °C until the late-log phase. Starter cultures were inoculated into 100 ml fresh LB media supplemented with Tween 80 (0.05% v/v) and kanamycin (20 μg/ml) and cultivated until OD_600_ 1. Harvested cells (10 min at 3000*g*, 4 °C) were washed twice with 50 mM MOPS (pH 7.9) and resuspended in the same buffer in the ratio of 30 mg of cells (wet weight) per 240 μl of the final cell suspension. The cells were disrupted by 10 cycles (40 s, 6 m/s) at FastPrep-24 (MP Biomedicals) using lysing matrix B beads (MP Biomedicals). Lysates in the volume of 1100 μl were centrifuged at 23,000*g*, 20 min, 4 °C. The pellets were washed twice with 50 mM MOPS (pH 7.9) and resuspended into the volume of 91.6 μl, thereby obtaining the CEF. A total of 900 μl of 23,000*g* supernatant was ultracentrifuged at 100,000*g* for 1 h at 4 °C. The pellet was washed twice with buffer as before and resuspended into the volume of 75 μl, thereby obtaining the PMF. The supernatant was used as the cytosol fraction. However, it should be noted that both lysate and cytosol fractions are 3× less concentrated compared to the standard fractionation protocol described above. This was taken into consideration in preparation of the sample aliquots for SDS-PAGE analyses.

### Proteomic analyses

#### The sample preparation

To the samples from differential centrifugation fractionation of *Msmeg* (100 μg of proteins per sample; fractions from Experiments 3, 4 and 5 from Fig. S1*A*), urea was added to a 6.4 M final concentration, and they were reduced by adding 5 mM DTT (30 min, 60 °C). Subsequently, the samples were alkylated in the presence of 15 mM iodoacetamide (20 min, room temperature in dark). The modification reaction was quenched by an addition of 10 mM DTT. For protein digestion, 4 μg of modified sequencing grade trypsin (Promega) was used, and the samples were incubated overnight at 37 °C. The enzymatic reaction was stopped by 0.5% TFA; peptides were purified by a microtip C18 SPE and dried in the ConcentratorPlus (Eppendorf).

The samples from the sucrose density gradient (25 μg of proteins per sample) were filled up with 100 mM Tris–HCl pH 7.8 to the final volume of 100 μl. Reduction and alkylation were performed as mentioned in the previous paragraph. Next, SP3 protein cleanup protocol with Sera-Mag Carboxylate SpeedBeads (Cytiva) was used as described (57). Washed magnetic beads were added to the samples in the ratio of 10 μg beads per 1 μg protein. For protein digestion, 1 μg of modified sequencing grade trypsin (Promega) was used and the samples were incubated overnight at 37 °C. The reactions were stopped by 0.5% TFA.

#### Liquid chromatography-tandem mass spectrometry analyses

For LC-MS analyses, 1 μg of peptides per sample was loaded onto a nanotrap cartridge (PepMap100 C18, 300 μm × 5 mm, 5 μm particle size, Dionex) and separated with an analytical column (Acclaim PepMap C18, 75 μm × 500 mm, 3 μm particle size, Dionex) mounted into Nanospray Flex ion source for subcellular fractions from differential centrifugation and EASY-Spray C18 analytical column having integrated nanospray emitter (75 μm × 500 mm, 2 μm particle size, Thermo Fisher Scientific) mounted into EASY-Spray ion source for fractions from sucrose gradient. The peptides were separated using UltiMate 3000 RSLCnano system (Dionex) in 120 min gradient 3 to 43% of acetonitrile with 0.1% formic acid. Spectral datasets were collected by Orbitrap Elite mass spectrometer (Thermo Fisher Scientific) operating in the data-dependent mode using Top15 strategy for filtering precursor ions. Precursors were measured with a resolution 120,000, and fragments were obtained by the higher energy collisional dissociation fragmentation with normalized collision energy 25 and a resolution 15,000 (58). Datasets were processed by MaxQuant (version 1.6.17; https://www.maxquant.org) (59) with built-in Andromeda search engine. The specific parameters for searching were the following: carbamidomethylation (C) as permanent modification and oxidation (M) as variable modifications, up to 2 tryptic miscleavages, 20 ppm mass tolerance for precursors (before recalibration) and fragments, and 4.5 ppm for precursors (after recalibration), and active match between runs feature. Proteins were quantified using iBAQ indices (spectral counting-based quantification). The search was performed against protein databases *M. smegmatis* (ATCC 700084/mc^2^155): uniprotkb_proteome_UP000000757_2023_10_20 (6602 sequences, UniProt, downloaded 20.10.2023).

## Data availability

The mass spectrometry proteomics data (Dataset 1) have been deposited to the ProteomeXchange Consortium *via* the PRIDE (60) partner repository with the dataset identifier PXD046591 and https://doi.org/10.6019/PXD046591.

## Supporting information

This article contains supporting information.

## Conflict of interest

The authors declare that they have no conflicts of interest with the contents of this article.
