# Supplementary material for: Compartmentalization of galactan biosynthesis in mycobacteria
Source: J Biol Chem. 2024 Feb 16;300(3):105768. doi: 10.1016/j.jbc.2024.105768 (PMC10951656; doi:10.1016/j.jbc.2024.105768)
Supplement: Supplemental Data and Figures S1–S8 [file mmc1.pdf]

SUPPORTING INFORMATION

Compartmentalization of galactan biosynthesis in mycobacteria

Karin Savková[1], Maksym Danchenko[2], Viktória Fabianová[1], Jana Bellová[2], Mária Bencúrová[1], Stanislav Huszár[1], Jana Korduláková[1], Barbara Siváková[2], Peter Baráth[2], Katarína Mikušová[1]*

[1]Department of Biochemistry, Faculty of Natural Sciences, Comenius University in Bratislava, Bratislava, Slovakia
[2]Institute of Chemistry, Slovak Academy of Sciences, Bratislava, Slovakia

LIST OF CONTENTS

Supplemental Data

Dataset 1: List of identified proteins.

Supplemental Figures

Figure S1: Characterization of *Msmeg* subcellular fractions obtained by differential centrifugation – additional data.

Figure S2: Distribution of recombinant GalE1, WbbL1, GlfT1 and GlfT2 in subcellular fractions.

Figure S3: Distribution of galactan activities in the whole cells and subcellular fractions of *Msmeg/*pVV2.

Figure S4: Examination of the effects of the cell disruption methods on the lipid composition and enzyme activities of subcellular fractions.

Figure S5: Characterization of *Msmeg* fractions obtained by separation on the sucrose gradient – additional data.

Figure S6: Extract for the proteomic analysis of the fractions obtained by separation on the 20-50% sucrose density gradient.

Figure S7: Production of [^{14}C]-LLG and their glycolipid precursors by fractions from sucrose gradient fractionation.

Figure S8. List of primers used for the construction of *Msmeg* strains overproducing GalE1 (MSMEG_6142), WbbL (MSMEG_1826), GlfT1 (MSMEG_6367) and GlfT2 (MSMEG_6403).

SUPPLEMENTAL DATA

Dataset 1: **List of identified proteins. A.** Subcellular fractions obtained by differential centrifugation of *Msmeg* lysates. The dataset (2735 proteins) includes identifiers from UniProt, protein names, sequence coverage (SC), theoretical molecular weight (MW), Andromeda score reflecting reliability of identification, number of matched fragmentation spectra, and iBAQ quantification values for each biological replicate of lysate (LYS), cell envelope fraction (CEF), plasma membrane fraction (PMF), and cytosol (CYT). The three sets of fractions labelled 42, 44 and 45 correspond to Experiment 3, Experiment 4 and Experiment 5, respectively, specified in Fig. S1A. **B**. Fractions obtained by separation of the total *Msmeg* cell lysate on the 20-50% sucrose density gradient, iBAQ quantification values for fractions L1-L12 are shown (2441 proteins). **C.** Fractions obtained by separation of the supernatant from the low-speed centrifugation of the *Msmeg* lysate on the 20-50% sucrose density gradient, iBAQ quantification values for fractions S1-S12 are shown (2416 proteins).

SUPPLEMENTAL FIGURES

A

Protein concentration in the fractions (µg/µl)

	LYS	CYT	PMF	CEF
**Experiment 1**	26.9	22.9	2.1	2.6
**Experiment 2**	23.1	20.8	1.7	1.9
**Experiment 3**	24.1	23.0	2.1	2.9
**Experiment 4**	26.6	22.1	1.7	2.3
**Experiment 5**	25.3	18.0	1.7	2.9

Distribution of lysate proteins in the fractions (%)

	100% (CYT+PMF+CEF)	CYT	PMF	CEF	
**Experiment 1**	27.6 µg/µl	83	8	9	100%
**Experiment 2**	24.3 µg/µl	85	7	8	100%
**Experiment 3**	27.9 µg/µl	82	7	10	100%
**Experiment 4**	26.1 µg/µl	85	6	9	100%
**Experiment 5**	22.6 µg/µl	80	7	13	100%
	Average	***83 ± 1 %***	***7 ± 0.2 %***	***10 ± 2 %***	

B

Pathway	Short name	UniProt ID	Mycobrowser ID	Mean iBAQ LYS	CEF	PMF	CYT
galactan biosynthesis and export	GalE1	A0R5C5	MSMEG_6142	1.6E+07	3.3E+05	6.9E+04	6.5E+06
	Glf	A0R629	MSMEG_6404	1.4E+07	1.3E+05	1.2E+04	6.4E+06
	WbbL	A0QTF9	MSMEG_1826	1.6E+06	2.8E+06	2.5E+06	4.0E+05
	GlfT1	A0R5Z2	MSMEG_6367	1.4E+06	2.7E+06	1.5E+06	2.3E+05
	GlfT2	A0R628	MSMEG_6403	1.6E+07	1.7E+07	1.3E+07	3.7E+06
	Wzt	A0R5Z1	MSMEG_6366	7.4E+06	2.3E+06	2.6E+06	1.1E+06
	Wzm	A0R5Z3	MSMEG_6369	0.0E+00	3.9E+05	5.2E+05	0.0E+00
respiration NADH oxidase	Ndh	A0QYD6	MSMEG_3621	6.4E+06	1.3E+07	8.6E+06	9.2E+05
PIM and LAM biosynthesis	LpqW	A0R2I8	MSMEG_5130	3.5E+05	3.7E+05	3.2E+05	5.1E+04
	PatA	A0QWG5	MSMEG_2934	2.6E+05	2.1E+05	5.9E+04	3.2E+04
	PimA	A0QWG6	MSMEG_2935	4.1E+05	7.7E+03	1.7E+05	1.3E+05
	PimB	A0R043	MSMEG_4253	1.8E+04	1.1E+05	2.7E+05	7.5E+03
	Ppm1	A0QZ12	MSMEG_3859	1.3E+07	2.0E+07	6.0E+06	2.5E+06
other pathways	Ger reduct	A0QR89	MSMEG_1028/2308	4.0E+07	4.5E+07	3.0E+07	7.5E+06
	Gtf1	A0QPG4	MSMEG_0389	2.9E+06	3.0E+06	3.8E+06	1.7E+05
	Membrane prot	A0QTS2	MSMEG_1944	9.2E+05	2.2E+05	8.1E+05	2.0E+05
	PyrD	A0QZY9	MSMEG_4198	1.2E+06	1.2E+06	1.5E+06	2.1E+05
	Wag31	A0R006	MSMEG_4217	1.9E+08	1.2E+08	1.2E+07	5.0E+07
	Psd	A0QQS6	MSMEG_0861	4.5E+06	3.9E+06	4.2E+06	7.9E+05
mycomembrane-associated	Ag85A	A0R623	MSMEG_6398	3.4E+07	3.6E+07	1.1E+06	5.8E+06
	Ag85C	A0QY95	MSMEG_3580	1.7E+07	9.9E+07	1.9E+06	3.6E+06
	Ag85C	A0R624	MSMEG_6399	8.0E+06	9.3E+06	1.5E+04	1.6E+06
	Ag85B	A0QU51	MSMEG_2078	2.6E+07	7.1E+07	8.5E+05	3.4E+06
	MspA/MspB	A0QR29/A0QPU4	MSMEG_0965/0520	6.3E+07	1.1E+08	8.5E+05	1.6E+06
	Mce1b	A0QNR3	MSMEG_0135	2.9E+05	1.5E+06	4.8E+04	0.0E+00
	Mce1c	A0QNR4	MSMEG_0136	3.0E+05	2.2E+06	1.5E+05	0.0E+00
	MceG	A0QS64	MSMEG_1366	2.9E+06	9.3E+06	1.4E+06	4.0E+05

C

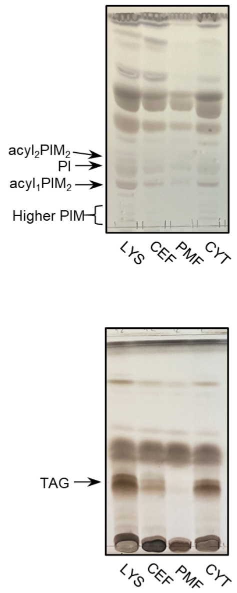

Figure S1: **Characterization of *Msmeg* subcellular fractions obtained by differential centrifugation – additional data**. **A.** Quantification of proteins in subcellular fractions from 5 different experiments. To show the distribution of the proteins among the fractions, the values for PMF and CEF are one quarter of the actual concentrations of the fractions prepared as described in the Methods section, as these were made as four times concentrated. Average values with standard deviations are shown. **B.** Mean iBAQ quantification values coded in red-yellow-green (high-medium-low) scale for each detected protein (with UniProt and Mycobrowser identifiers) characteristic for selected pathways (GalE1 – UDP-glucose 4-epimerase, Glf – UDP-galactopyranose mutase, WbbL – rhamnosyl transferase, GlfT1 – initiating galactosyl transferase, GlfT2 – polymerizing galactosyl transferase, Wzt – ATP-binding protein of an ABC transporter of lipid-linked galactan, Wzm – integral membrane protein of an ABC transporter of lipid-linked galactan, Ndh – NADH dehydrogenase, LpqW – monoacyl phosphatidylinositol tetramannoside-binding protein, PatA – phosphatidylinositol mannoside acyltransferase, PimA – phosphatidyl-myo-inositol mannosyltransferase, PimB – GDP-mannose-dependent α-(1-6)-phosphatidylinositol monomannoside mannosyltransferase, Ppm1 – polyprenol-monophosphomannose synthase, Ger reduct – geranylgeranyl reductase, Gtf1 – glycosyl transferase, Membrane prot – membrane protein, PyrD – dihydroorotate dehydrogenase, Wag31 – DivIVA protein, Psd – phosphatidylserine decarboxylase proenzyme, Ag85A/C – Antigen 85-A/C, Ag85B – antigen 85-B, MspA/B – porin, Mce1b/c – MCE family protein, MceG – ATP-binding cassette transporter, LAM – lipoarabinomannan). Data from three independent biological replicates are shown in Dataset 1. **C.** TLC analysis of the lipid aliquots separated in in $CHCl_3/CH_3OH/NH_4OH/H_2O$ (65:25:0.5:4) (*upper panel*) or petroleum ether (60–80°C)/ethyl acetate (98:2, 3 runs) (*bottom panel*). The lipids were visualized with the cupric sulfate reagent. Representative images from at least three independent biological replicates are shown. LYS – lysate, CEF – cell envelope fraction, PMF – plasma membrane fraction, CYT – cytosol, PI – phosphatidyl inositol, PIM – phosphatidyl inositol mannosides, TAG – triacylglycerols.

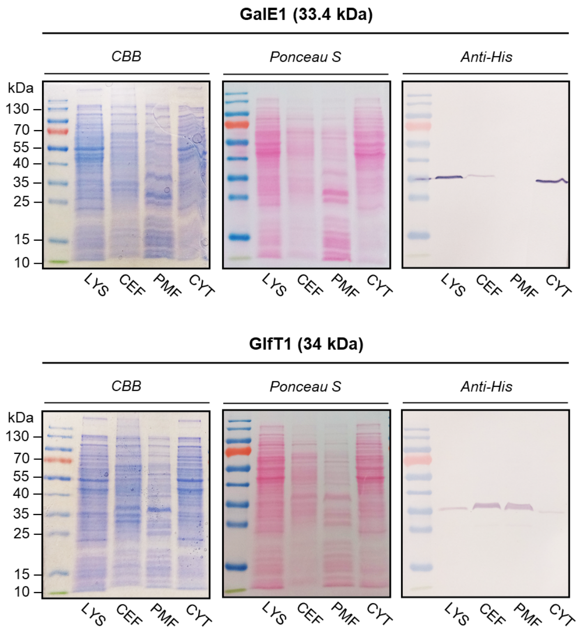

Figure S2: **Distribution of recombinant GalE1, WbbL1, GlfT1 and GlfT2 in subcellular fractions.** Aliquot volumes of the samples were analyzed, but the lysate and cytosol fractions were 10 × diluted. Proteins were visualized by staining with Coomassie Brilliant Blue (CBB) and Ponceau S. Recombinant proteins were immunodetected with anti-His antibodies and alkaline phosphatase-conjugated secondary antibodies. LYS – lysate, CEF – cell envelope fraction, PMF – plasma membrane fraction, CYT – cytosol.

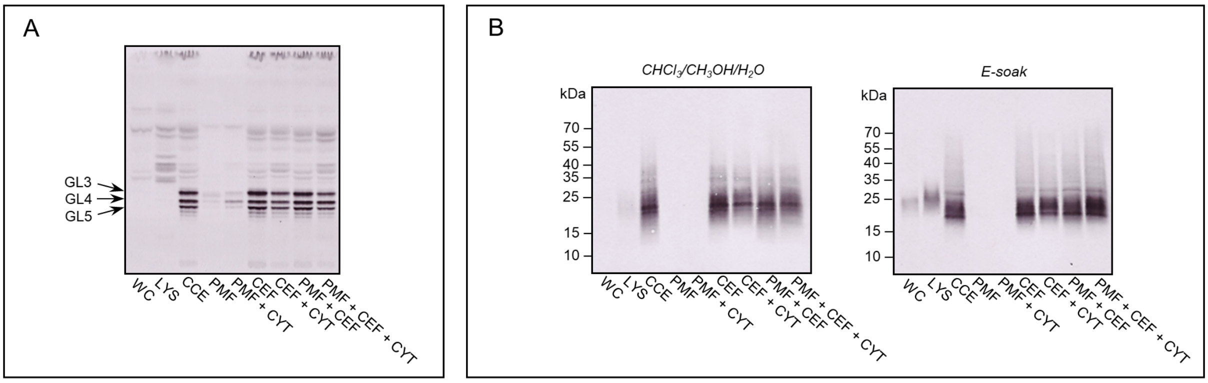

Figure S3: **Distribution of galactan activities in the whole cells and subcellular fractions of *Msmeg/*pVV2**. **A.** TLC analysis of [^{14}C]-labelled glycolipids separated in $CHCl_3/CH_3OH$/1 M ammonium acetate/NH_4OH/H_2O (180:140:9:9:23). **B.** Analysis of the [^{14}C]-LLG from $CHCl_3/CH_3OH/H_2O$ (10:10:3) and E-soak [H_2O/C_2H_5OH/diethyl ether/pyridine/NH_4OH (15:15:5:1:0.017)] extracts by SDS-PAGE followed by the transfer to a nitrocellulose membrane. Reaction products were visualized by autoradiography. WC – whole cells; LYS – lysate, CCE – complete cell envelope (100,000 × g pellet of the lysate), PMF – plasma membrane fraction, CEF – cell envelope fraction, CYT – cytosol, GL – glycolipid.

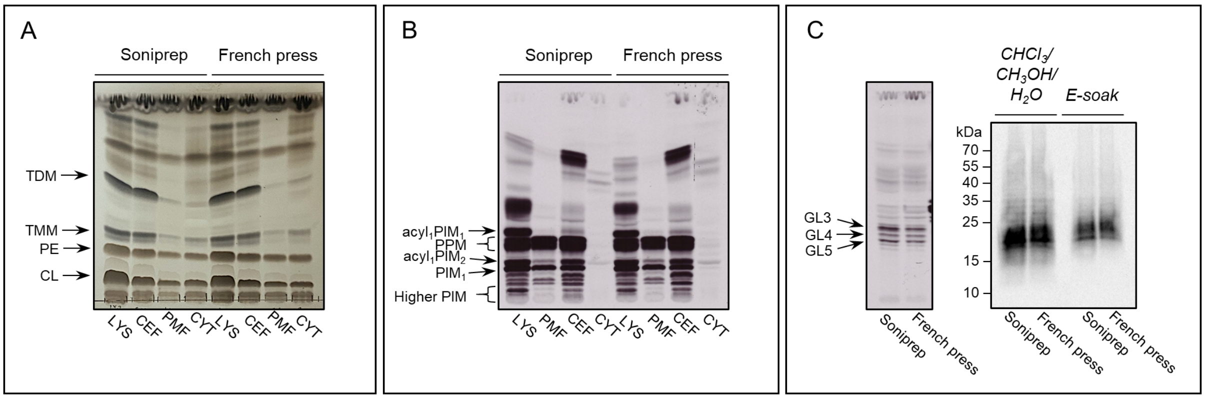

Figure S4: **Examination of the effects of the cell disruption methods on the lipid composition and enzyme activities of subcellular fractions.** *Msmeg* cells were disrupted by sonication or French press. Subcellular fractions were prepared from lysates by differential centrifugation, as described. **A.** TLC analysis of the lipid aliquots separated in $CHCl_3/CH_3OH/H_2O$ (20:4:0.5). The lipids were visualized with the cupric sulfate reagent. **B.** [^{14}C]-mannolipid synthesis. TLC analysis of products from enzyme reactions performed with the fraction aliquots and GDP-[^{14}C] Man. The extracted lipids were analyzed by TLC in $CHCl_3/CH_3OH/NH_4OH/H_2O$ (65:25:0.5:4) **C.** Production of the lipid-linked [^{14}C]-galactan and its precursors by CEF fractions. Reaction mixtures were supplemented with NADH, UDP-GlcNAc, TDP-Rha and UDP-[^{14}C]-Gal*p*, and spiked with cytosol. *Left panel:* TLC analysis of [^{14}C]-labeled glycolipids separated in $CHCl_3/CH_3OH$/1 M ammonium acetate/NH_4OH/H_2O (180:140:9:9:23). *Right panel:* Analysis of the [^{14}C]-LLG from $CHCl_3/CH_3OH/H_2O$ (10:10:3) and E-soak [H_2O/C_2H_5OH/diethyl ether/pyridine/NH_4OH (15:15:5:1:0.017)] extracts by SDS-PAGE followed by the transfer to a nitrocellulose membrane. The radiolabeled reaction products were visualized by autoradiography or phosphor imaging. LYS – lysate, CEF – cell envelope fraction, PMF – plasma membrane fraction, CYT – cytosol, TDM – trehalose dimycolates, TMM – trehalose monomycolates, PE – phosphatidyl ethanolamine, CL – cardiolipin, PIM – phosphatidylinositol mannosides, PPM – polyprenylphosphomannoses, GL – glycolipid, LLG – lipid-linked galactan.

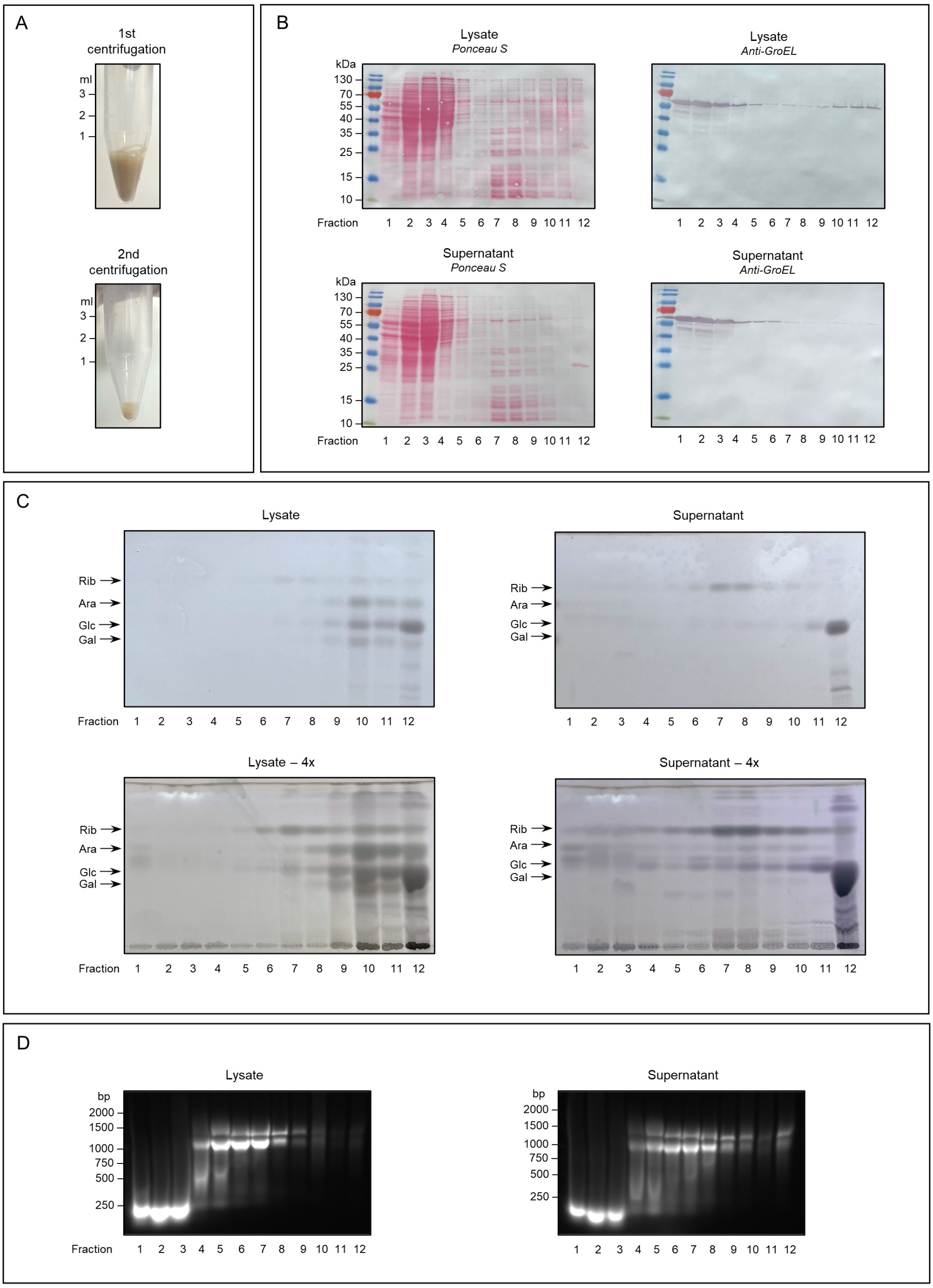

Figure S5: **Characterization of *Msmeg* fractions obtained by separation on the sucrose gradient – additional data. A.** Sediments obtained by low-speed centrifugation of 3 ml of cell lysate. **B.** Protein analysis by SDS-PAGE and Western blotting. Aliquot volumes (10 µl) of each fraction were analyzed. *Left panels*: Proteins were visualized with Ponceau S; *right panels:* Immunodetection with anti-GroEL2 antibodies and alkaline phosphatase-conjugated secondary antibodies. **C.** Monosaccharide composition of insoluble pellets. The pellets were hydrolyzed with 2 M trifluoroacetic acid, and the released monosaccharides were separated by TLC in ethyl acetate/pyridine/glacial acetic acid/H_2O (6:3:1:1). The monosaccharides were visualized with α-naphthol stain. **D**: Nucleic acid analysis. 10 µl from each fraction were analyzed by standard agarose gel electrophoresis. Ara – arabinose, Gal – galactose, Glc – glucose, Rib – ribose.

				CYT				PMF				CEF			
Fraction				1	2	3	4	5	6	7	8	9	10	11	12
				Lysate											
Normalisation factor				35	13	15	29	82	87	67	75	81	68	80	106
galactan biosynthesis and export	GalE1	A0R5C5	MSMEG_6142	3.5E+05	9.9E+05	5.3E+05	1.6E+05	6.3E+03	2.3E+03	2.9E+03	8.6E+02	2.3E+03	7.1E+03	2.4E+04	1.2E+04
	Glf	A0R629	MSMEG_6404	6.1E+04	2.4E+05	1.3E+05	5.0E+04	2.5E+03	0.0E+00	6.7E+02	1.6E+02	1.9E+02	1.9E+03	6.3E+03	3.1E+03
	WbbL	A0QTF9	MSMEG_1826	0.0E+00	0.0E+00	0.0E+00	4.8E+02	1.1E+04	2.0E+04	1.6E+04	8.0E+03	2.0E+04	2.3E+04	2.1E+04	1.0E+04
	GlfT1	A0R5Z2	MSMEG_6367	0.0E+00	0.0E+00	0.0E+00	1.2E+02	9.2E+03	1.4E+04	1.6E+04	7.2E+03	1.2E+04	1.7E+04	1.6E+04	5.7E+03
	GlfT2	A0R628	MSMEG_6403	1.6E+02	6.4E+02	3.3E+02	5.0E+03	6.5E+04	1.2E+05	8.5E+04	4.0E+04	5.5E+04	7.2E+04	7.5E+04	3.3E+04
	Wzt	A0R5Z1	MSMEG_6366	6.1E+03	1.6E+04	1.0E+04	8.5E+03	3.1E+03	1.5E+03	2.1E+03	1.8E+03	5.4E+03	7.4E+03	7.6E+03	4.0E+03
	Wzm	A0R5Z3	MSMEG_6369	0.0E+00	0.0E+00	0.0E+00	1.2E+03	9.5E+02	1.2E+03	1.8E+03	3.2E+03	7.1E+03	6.2E+03	5.2E+03	2.4E+03
respiration NADH oxidase	Ndh	A0QYD6	MSMEG_3621	1.0E+03	6.0E+03	1.1E+04	1.9E+04	3.9E+04	7.4E+04	7.6E+04	8.3E+04	1.9E+05	2.4E+05	1.8E+05	9.3E+04
PIM and LAM biosynthesis	LpqW	A0R2I8	MSMEG_5130	0.0E+00	0.0E+00	0.0E+00	7.1E+01	0.0E+00	7.5E+02	5.3E+02	1.9E+03	4.9E+03	6.0E+03	5.1E+03	2.0E+03
	PatA	A0QWG5	MSMEG_2934	6.1E+02	8.1E+03	7.9E+03	7.3E+03	9.3E+03	7.3E+03	8.0E+03	4.0E+03	4.9E+03	5.9E+03	6.8E+03	2.1E+03
	PimB	A0R043	MSMEG_4253	0.0E+00	0.0E+00	4.0E+03	1.8E+03	3.2E+03	6.8E+03	5.6E+03	1.8E+03	3.0E+03	3.9E+03	2.5E+03	1.3E+03
	Ppm1	A0QZ12	MSMEG_3859	5.7E+03	2.7E+04	3.3E+04	1.8E+04	3.0E+04	2.7E+04	2.9E+04	1.8E+04	3.6E+04	4.5E+04	4.5E+04	2.1E+04
other pathways	Ger reduct	A0QR89	MSMEG_1028/2308	5.4E+03	3.0E+04	6.3E+04	7.4E+04	1.9E+05	3.5E+05	2.4E+05	1.2E+05	1.5E+05	2.0E+05	2.0E+05	9.8E+04
	Gtf1	A0QPG4	MSMEG_0389	1.1E+03	0.0E+00	0.0E+00	0.0E+00	4.8E+03	8.6E+03	7.7E+03	6.0E+03	7.6E+03	1.2E+04	9.4E+03	7.2E+03
	Membrane prot	A0QTS2	MSMEG_1944	0.0E+00	0.0E+00	0.0E+00	0.0E+00	4.8E+03	1.1E+04	6.8E+03	3.0E+03	3.3E+03	4.4E+03	4.1E+03	2.4E+03
	PyrD	A0QZY9	MSMEG_4198	0.0E+00	0.0E+00	0.0E+00	3.6E+02	6.8E+03	1.7E+04	9.4E+03	6.1E+03	9.1E+03	9.5E+03	9.4E+03	5.0E+03
	Wag31	A0R006	MSMEG_4217	2.7E+05	1.3E+05	4.8E+04	9.8E+04	2.8E+04	4.5E+03	6.7E+03	1.2E+04	8.5E+04	1.3E+05	1.4E+05	1.2E+05
	Psd	A0QQS6	MSMEG_0861	0.0E+00	0.0E+00	0.0E+00	4.0E+03	1.6E+04	4.6E+04	2.6E+04	1.1E+04	1.2E+04	2.4E+04	1.6E+04	1.4E+04
mycomembrane-associated	Ag85A	A0R623	MSMEG_6398	7.3E+05	1.7E+06	7.0E+05	2.1E+05	7.5E+03	3.3E+03	2.4E+04	1.1E+05	4.3E+05	5.2E+05	4.7E+05	2.4E+05
	Ag85C	A0QY95	MSMEG_3580	2.7E+05	6.1E+05	1.8E+05	5.8E+04	1.6E+03	1.3E+03	1.8E+04	1.0E+05	3.6E+05	6.9E+05	5.3E+05	2.1E+05
	Ag85C	A0R624	MSMEG_6399	9.9E+04	1.9E+05	8.3E+04	2.1E+04	0.0E+00	0.0E+00	1.6E+03	5.9E+03	2.6E+04	3.5E+04	3.5E+04	1.8E+04
	Ag85B	A0QU51	MSMEG_2078	3.0E+05	8.7E+05	2.8E+05	1.1E+05	2.6E+03	1.3E+03	1.8E+04	8.6E+04	2.4E+05	3.1E+05	2.8E+05	1.2E+05
	MspA/MspB	A0QR29/A0QPU4	MSMEG_0965/0520	2.0E+05	4.1E+05	1.2E+05	4.4E+04	0.0E+00	1.0E+03	9.6E+03	2.5E+04	9.1E+04	1.6E+05	1.2E+05	7.2E+04
	Mce1b	A0QNR3	MSMEG_0135	4.3E+02	0.0E+00	0.0E+00	0.0E+00	0.0E+00	1.9E+02	1.6E+03	4.4E+03	1.9E+04	2.1E+04	2.2E+04	9.8E+03
	Mce1c	A0QNR4	MSMEG_0136	4.5E+03	1.7E+04	1.1E+04	5.4E+03	0.0E+00	3.6E+02	1.1E+03	2.9E+03	1.7E+04	2.2E+04	1.4E+04	8.7E+03
	MceG	A0QS64	MSMEG_1366	1.7E+04	8.3E+04	7.3E+04	4.3E+04	1.5E+04	1.4E+04	2.2E+04	4.5E+04	1.4E+05	1.5E+05	1.3E+05	6.7E+04
				Supernatant											
Normalisation factor				49	15	13	51	99	149	74	77	91	100	328	222
galactan biosynthesis and export	GalE1	A0R5C5	MSMEG_6142	2.8E+05	8.7E+05	6.7E+05	3.5E+04	9.7E+03	2.8E+03	1.7E+03	1.1E+03	9.2E+02	1.7E+03	6.6E+02	9.0E+02
	Glf	A0R629	MSMEG_6404	4.2E+04	1.9E+05	1.6E+05	1.2E+04	4.2E+03	1.0E+03	3.2E+02	0.0E+00	0.0E+00	2.1E+02	1.9E+02	1.1E+02
	WbbL	A0QTF9	MSMEG_1826	1.2E+03	0.0E+00	0.0E+00	1.5E+03	1.0E+04	1.7E+04	1.2E+04	8.3E+03	6.6E+03	6.4E+03	3.5E+03	3.8E+03
	GlfT1	A0R5Z2	MSMEG_6367	0.0E+00	0.0E+00	0.0E+00	1.7E+02	8.6E+03	1.9E+04	1.1E+04	4.6E+03	4.6E+03	4.2E+03	3.0E+03	2.2E+03
	GlfT2	A0R628	MSMEG_6403	1.1E+02	5.0E+03	1.5E+02	6.9E+03	7.1E+04	1.1E+05	8.0E+04	3.5E+04	2.5E+04	1.6E+04	9.4E+03	1.0E+04
	Wzt	A0R5Z1	MSMEG_6366	3.5E+03	8.9E+03	7.4E+03	3.4E+03	3.5E+03	1.5E+03	5.9E+02	1.0E+03	2.0E+03	2.6E+03	2.1E+03	1.5E+03
	Wzm	A0R5Z3	MSMEG_6369	0.0E+00	0.0E+00	0.0E+00	0.0E+00	9.7E+02	1.5E+03	1.5E+03	2.1E+03	4.1E+03	3.4E+03	2.0E+03	2.4E+03
respiration NADH oxidase	Ndh	A0QYD6	MSMEG_3621	3.2E+03	0.0E+00	1.1E+04	1.6E+04	5.1E+04	5.9E+04	7.7E+04	7.8E+04	1.0E+05	1.1E+05	4.6E+04	3.6E+04
PIM and LAM biosynthesis	LpqW	A0R2I8	MSMEG_5130	0.0E+00	0.0E+00	0.0E+00	1.3E+02	0.0E+00	2.7E+02	2.0E+02	4.3E+02	5.6E+02	7.2E+02	4.4E+02	2.5E+02
	PatA	A0QWG5	MSMEG_2934	1.4E+03	7.3E+03	1.8E+04	7.2E+03	7.1E+03	4.7E+03	5.2E+03	2.7E+03	3.1E+03	1.6E+03	7.5E+02	1.1E+03
	PimB	A0R043	MSMEG_4253	7.1E+02	0.0E+00	0.0E+00	0.0E+00	3.3E+03	5.6E+03	4.5E+03	1.4E+03	2.6E+02	6.4E+02	2.0E+02	1.5E+02
	Ppm1	A0QZ12	MSMEG_3859	1.2E+04	1.3E+04	1.8E+04	1.2E+04	2.2E+04	1.6E+04	2.0E+04	1.7E+04	1.5E+04	1.3E+04	8.6E+03	6.2E+03
other pathways	Ger reduct	A0QR89	MSMEG_1028/2308	1.1E+04	2.3E+04	6.4E+04	6.3E+04	2.5E+05	3.1E+05	2.1E+05	1.1E+05	1.0E+05	6.4E+04	4.3E+04	3.9E+04
	Gtf1	A0QPG4	MSMEG_0389	1.4E+03	3.8E+03	0.0E+00	1.1E+03	6.5E+03	8.9E+03	5.9E+03	4.9E+03	3.1E+03	3.7E+03	1.8E+03	1.4E+03
	Membrane prot	A0QTS2	MSMEG_1944	0.0E+00	0.0E+00	0.0E+00	0.0E+00	6.6E+03	1.0E+04	1.2E+04	5.8E+03	3.4E+03	2.2E+03	1.3E+03	1.2E+03
	PyrD	A0QZY9	MSMEG_4198	0.0E+00	0.0E+00	0.0E+00	1.0E+03	9.1E+03	1.4E+04	9.9E+03	5.7E+03	3.7E+03	3.6E+03	2.3E+03	2.2E+03
	Wag31	A0R006	MSMEG_4217	4.8E+05	1.8E+05	3.2E+04	4.8E+04	2.7E+04	7.3E+03	3.2E+03	4.9E+03	1.1E+04	3.9E+04	2.4E+04	1.5E+04
	Psd	A0QQS6	MSMEG_0861	0.0E+00	0.0E+00	0.0E+00	2.7E+03	2.0E+04	4.7E+04	1.8E+04	9.7E+03	8.3E+03	2.8E+03	2.1E+03	3.3E+03
mycomembrane-associated	Ag85A	A0R623	MSMEG_6398	7.7E+05	1.5E+06	6.8E+05	5.4E+04	2.0E+04	4.7E+03	3.3E+03	9.5E+03	1.1E+04	9.5E+03	1.8E+03	2.4E+03
	Ag85C	A0QY95	MSMEG_3580	1.7E+05	2.5E+05	1.8E+05	7.2E+03	2.1E+03	0.0E+00	2.0E+03	2.9E+03	7.5E+03	5.7E+03	0.0E+00	0.0E+00
	Ag85C	A0R624	MSMEG_6399	9.7E+04	1.4E+05	8.8E+04	1.7E+03	8.3E+02	2.3E+02	0.0E+00	0.0E+00	3.9E+02	4.2E+02	0.0E+00	0.0E+00
	Ag85B	A0QU51	MSMEG_2078	2.3E+05	3.7E+05	2.7E+05	1.3E+04	3.4E+03	9.7E+02	1.7E+03	6.7E+03	8.2E+03	4.1E+03	3.7E+02	4.2E+02
	MspA/MspB	A0QR29/A0QPU4	MSMEG_0965/0520	2.1E+05	3.5E+05	1.9E+05	1.5E+04	1.9E+03	1.8E+03	7.6E+02	3.4E+03	7.2E+03	5.1E+03	4.4E+02	0.0E+00
	Mce1b	A0QNR3	MSMEG_0135	0.0E+00	0.0E+00	0.0E+00	0.0E+00	1.8E+02	1.4E+02	2.0E+02	3.0E+02	3.9E+02	4.9E+02	8.5E+01	0.0E+00
	Mce1c	A0QNR4	MSMEG_0136	3.5E+03	9.4E+03	1.0E+04	5.9E+02	5.9E+02	1.9E+02	3.7E+01	1.0E+02	2.2E+02	0.0E+00	0.0E+00	3.4E+01
	MceG	A0QS64	MSMEG_1366	7.5E+03	4.4E+04	8.1E+04	1.8E+04	1.6E+04	1.1E+04	1.3E+04	2.7E+04	4.1E+04	3.0E+04	2.1E+04	1.5E+04

Figure S6: **Extract for the proteomic analysis of the fractions obtained by separation on the 20-50% sucrose density gradient.** Volume-normalized iBAQ quantification values distribution among fractions 1-12 color-coded in red-yellow-green (high-medium-low) scale for each detected protein (with UniProt and Mycobrowser identifiers) characteristic for selected pathways (GalE1 – UDP-glucose 4-epimerase, Glf – UDP-galactopyranose mutase, WbbL – rhamnosyl transferase, GlfT1 – initiating galactosyl transferase, GlfT2 – polymerizing galactosyl transferase, Wzt – ATP-binding protein of an ABC transporter of lipid-linked galactan, Wzm – integral membrane protein of an ABC transporter of lipid-linked galactan, Ndh – NADH dehydrogenase, LpqW – monoacyl phosphatidylinositol tetramannoside-binding protein, PatA – phosphatidylinositol mannoside acyltransferase, PimB – GDP-mannose-dependent α-(1-6)-phosphatidylinositol monomannoside mannosyltransferase, Ppm1 – polyprenol-monophosphomannose synthase, Ger reduct – geranylgeranyl reductase, Gtf1 – glycosyl transferase, Membrane prot – membrane protein, PyrD – dihydroorotate dehydrogenase, Wag31 – DivIVA family protein, Psd – phosphatidylserine decarboxylase, Ag85A/C – Antigen 85-A/C, Ag85B – antigen 85-B, MspA/B – porin, Mce1b/c – MCE family protein, MceG – ATP-binding cassette transporter, LAM – lipoarabinomannan, PIM – phosphatidyl-myo-inositol mannoside, CYT – cytosol, PMF – plasma membrane fraction, CEF – cell envelope fraction).

A

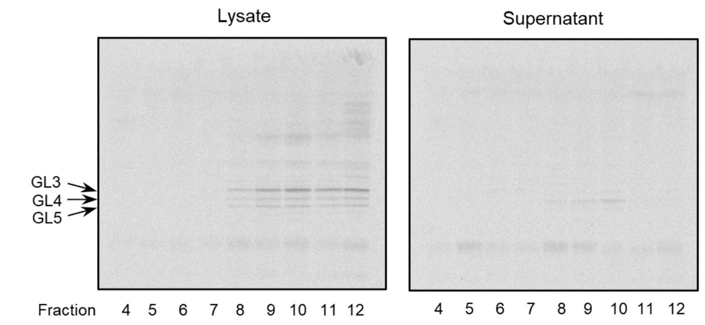

B

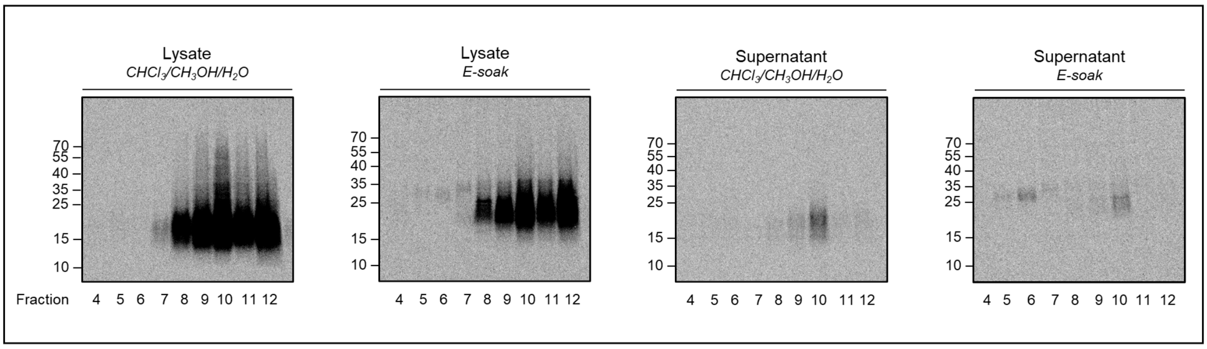

Figure S7: **Production of [^{14}C]-LLG and their glycolipid precursors by fractions from sucrose gradient fractionation. A.** TLC analysis of [^{14}C]-labelled LLG-precursors. Solvent: $CHCl_3$/CH_3OH/1 M ammonium acetate/NH_4OH/H_2O (180:140:9:9:23). GL3-5 – glycolipid 3-5 (decaprenyl-P-P-GlcNAc-Rha-[^{14}C]-Gal_{1-3}). **B.** [^{14}C]-LLG analysis**.** The signals from Fig. 4C were amplified by longer exposure. Fractions 4-12 from sucrose gradients served as the source of acceptor substrates and enzymes in the reaction mixtures, which were supplemented with UDP-GlcNAc, TDP-Rha and UDP-[^{14}C]-Gal*p*.
[^{14}C]-LLGs extracted by $CHCl_3$/CH_3OH/H_2O (10:10:3) and E-soak [H_2O/C_2H_5OH/diethyl ether/pyridine/NH_4OH (15:15:5:1:0.017)] were analyzed by SDS-PAGE, followed by the transfer to a nitrocellulose membrane. Radioactive signals were detected by phosphor imaging. Identity of the weak signals present in E-soak fractions 5-7 is not known.

**Primer name**	**Sequence (5'→3')**
pVV2-MSMEG_6142-Fwd	AGT TTTACATATGGTGGCGGTGCGAACTTTGG
pVV2-MSMEG_6142-Rev	ACAAAGCTTTTACTGGGATTTGTTGCGGAAG
pVV2-MSMEG_1826-Fwd	AGTTTTACATATGGTGGTCGTGACGGTGACATAT
pVV2-MSMEG_1826-Rev	ACCAAGCTTTCACCACGCCCCTTTGTTCG
pVV2-MSMEG_6367-Fwd	GCACCAACATATGACGCACACTGAGGTCGTCTG
pVV2-MSMEG_6367-Rev	CCCAAGCTTTCATCGCTGGAACCTTTCGCGTC
pVV2-MSMEG_6403-Fwd	ATTCATATGAGTGACATCCCTTCCGGCGC
pVV2-MSMEG_6403-Rev	ATTAAGCTTTCATCGTCCGACTTTCTCCGG

Figure S8. **List of primers used for the construction of *Msmeg* strains overproducing GalE1 (MSMEG_6142), WbbL (MSMEG_1826), GlfT1 (MSMEG_6367) and GlfT2 (MSMEG_6403).**
